# Mechanically Tunable DNA Hydrogels as Prospective Biosensing Modules

**DOI:** 10.1002/marc.202500149

**Published:** 2025-05-08

**Authors:** Asya E. Can, Abdul W. U. Ali, Claude Oelschlaeger, Norbert Willenbacher, Iliya D. Stoev

**Affiliations:** ^1^ Institute of Biological and Chemical Systems ‐ Biological Information Processing Karlsruhe Institute of Technology 76344 Eggenstein‐Leopoldshafen Germany; ^2^ Institute for Mechanical Process Engineering and Mechanics Karlsruhe Institute of Technology 76131 Karlsruhe Germany

**Keywords:** biosensing, DNA materials, melting temperature, microrheology, sequence‐programmability

## Abstract

Sequence‐programmable DNA building blocks offer high degree of freedom in designing arbitrarily complex networks of tunable viscoelastic properties. Yet, the deployment of DNA‐based functional materials remains limited due to insufficient control over the emerging structures and their mechanics. In an ongoing effort to place structure‐property relations in stimuli‐responsive DNA materials on a firm foundation, here a systematic rheological study of self‐assembling DNA networks is presented, comprised of short DNA nanomotifs, namely trivalent nanostars and bivalent linkers, where the latter differ in their composition on a single base‐pair level. Notably, we found through combining conventional bulk rheology with diffusing wave spectroscopy (DWS‐based) passive microrheology a relationship between the melting temperature of a DNA hydrogel and its DNA sequence composition. By providing a use case, we demonstrated how the determination of such empirical relations could impact the areas of biosensing and mechanical computing, where control over the system state and target identification are key.

## Graphical abstract

Sequence‐programmable and thermally responsive DNA hydrogels enable highly sensitive mechanical biosensing through the exchange of linker strands using a toehold strand displacement mechanism. This new type of mechanical biosensor has in‐built sequence‐specificity and dose‐sensitivity, obviating the need for elaborate DNA sequencing and enabling rapid identification of target genomes within a diverse pool of other nucleic acids.

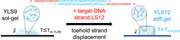

## 1 Introduction

The realisation in the 1990s that DNA can function as a “smart glue” to connect soft matter components into higher‐order assemblies marked the beginning of DNA nanotechnology.^[^ ^1^, ^2^, ^3^ ^]^ This field has been propelled by the sequence programmability of DNA oligomers, which bind according to strictly defined Watson–Crick base‐pairing rules.^[^ ^4^, ^5^ ^]^ This property has facilitated the design of functional materials that can sense and respond to external stimuli, e.g. temperature, pH, and light, enabling biosensing applications targeting the identification of DNA sequences and confirmation of genome integrity.^[^ ^6^, ^7^, ^8^, ^9^, ^10^, ^11^, ^12^, ^13^ ^]^

An emerging class of such materials is biocompatible DNA hydrogels,^[^ ^14^, ^15^ ^]^ which represent self‐assembled polymeric networks in a saline aqueous environment. These physically or chemically cross‐linked networks have mechanical properties determined by the base composition of the DNA strands.^[^ ^16^, ^17^, ^18^ ^]^ Nanomotifs constructed from short oligomers serve as customizable building blocks for complex 3D porous architectures with tailored, emergent mechanical and thermodynamic properties.^[^ ^19^, ^20^, ^21^ ^]^ Reversible physical interactions, mediated by hydrogen bonding, allow high tunability of the sol‐gel transition in multi‐purpose and multi‐use hydrogels, enabling their utility in early diagnostic applications where mechanical changes can signal the presence or degradation of a target DNA genome.

Advances in light scattering microrheology have facilitated efforts to quantify the mechanical response of such hydrogels concerning DNA sequence design.^[^ ^22^, ^23^, ^24^ ^]^ For instance, Xing *et al.* ^[^ ^7^ ^]^ used diffusing wave spectroscopy to identify and tune a sol‐gel transition in a suspension of Y‐shaped DNA particles with complementary sticky ends, demonstrating precise control over thermal stability through fine adjustments to linker length or the inclusion of free spacers. The critical role of inert, non‐binding joints and flexible linkers in gel formation has also been highlighted, revealing that increased flexibility weakens or even suppresses gelation.^[^ ^6^, ^25^ ^]^

**Figure 1:**
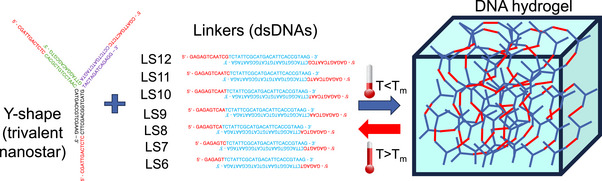
Schematic illustration of the formation of trivalent Y‐shapes and bivalent linkers with the DNA sequences shown in Table 1 of the Experimental Section. Combining the two building blocks at a well‐defined stoichiometry and low temperatures (below the melting temperature of the sticky ends in red) results in the formation of a DNA hydrogel. The latter is held together by hydrogen‐bonding interactions and can reversibly associate and dissociate by cycling the temperature on both sides of the melting transition.

Despite growing evidence confirming the high programmability and stimuli‐responsiveness of DNA‐based materials, a concrete link between DNA sequence design and material properties through scaling laws remains elusive. This study employs single‐base modifications in artificial DNA‐hydrogel assemblies (**Figure** **1**) to probe macroscopic structural details and inform the development of a comprehensive framework for designing DNA hydrogels with customizable properties. The generation of a library or catalogue of mechanically tunable and responsive materials is expected to provide innovative solutions to applications in precision diagnostics,^[^ ^26^ ^]^ dynamic scaffolds for stem cell adhesion,^[^ ^27^ ^]^ drug delivery systems,^[^ ^28^, ^29^ ^]^ tissue engineering,^[^ ^30^, ^31^, ^32^ ^]^ soft robotics,^[^ ^18^, ^33^ ^]^ adaptive materials,^[^ ^14^, ^34^ ^]^ and information processing platforms.^[^ ^35^ ^]^

## 2 Results and Discussion

### 2.1 Passive Microrheology of Mixed‐Valency DNA Hydrogels Via Diffusing Wave Spectroscopy

**Figure 2:**
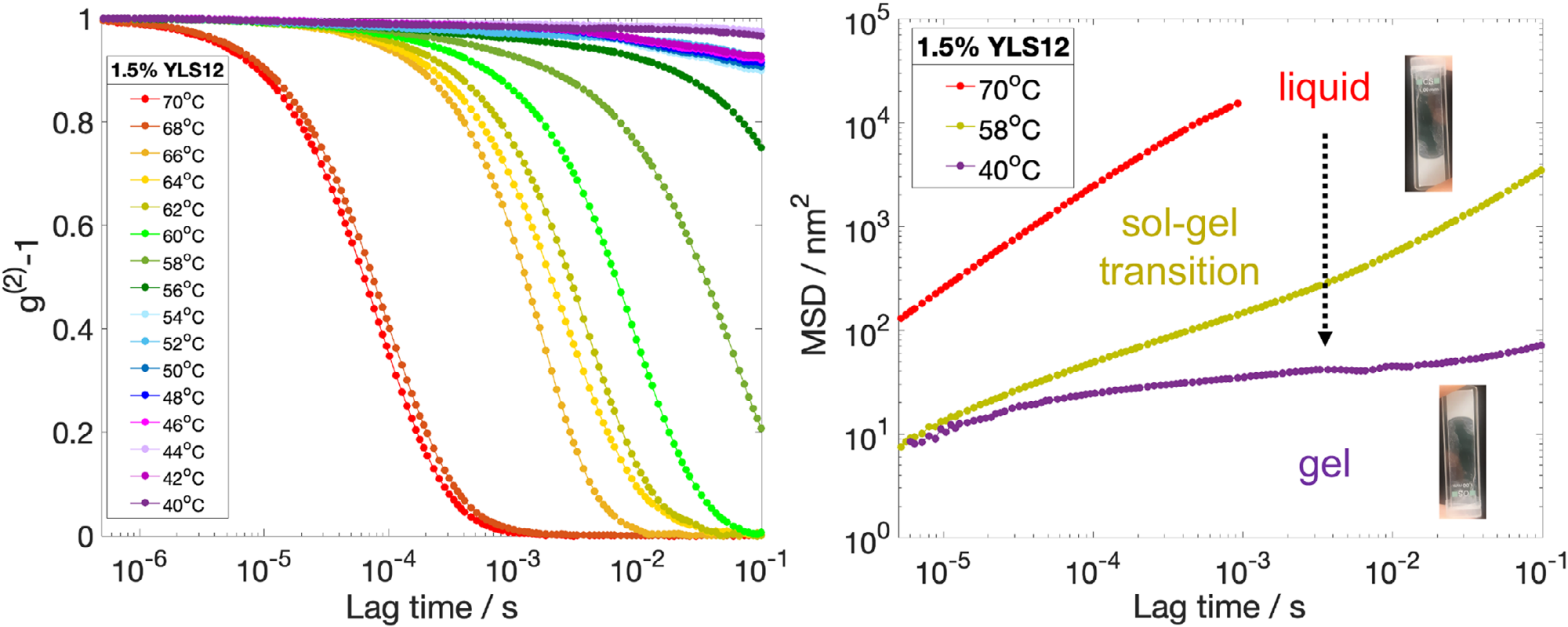
Passive microrheology using diffusing wave spectroscopy on 1.5 wt% YLS12: temperature ramp from 70°C to 40°C. We plot both the intensity autocorrelation functions g^(2)^‐1 and the mean‐squared displacements above, around and below the melting temperature of the system. The increasingly long decorrelation times and appearance of intermediate‐time plateau in the MSD both signify gelation.

As shown in Figure 1, self‐assembled bivalent DNA linkers (LS6‐12, with 6‐12‐base‐long sticky ends) were used as a bridge between trivalent DNA nanostars (Y‐shapes with 12‐base‐long sticky ends) to form a percolating hydrogel above a threshold concentration that ensures continuous sol‐to‐gel transition. To detect small differential viscoelastic response in these DNA hydrogels, we used diffusing wave spectroscopy (DWS) — a passive microrheology tool that has already successfully extracted dynamic mechanical information from similar complex fluids.^[^ ^7^, ^36^, ^37^, ^38^ ^]^ In these experiments, we used 260‐nm polystyrene tracer particles of 2 wt% final concentration and coated with polyethylene glycol to provide steric stabilization. **Figure** **2** shows the time evolution of the intensity autocorrelation function *g* ^(2)^ − 1 for 1.5 wt% (ca. 500 µm  DNA) YLS12 hydrogel upon gradual cooling from 70°C to 40°C. The fully dissociated system at high temperatures marked a progressive increase in the decorrelation time on cooling, where we found a transition to a more viscoelastic system triggered by the formation of the building blocks. At around 58°C we found an incomplete decay of the autocorrelation function over the measured time interval. Further cooling into the gel state caused the detected speckle pattern to remain nearly fully correlated over hundreds of milliseconds. This manifested into a plateau in the mean‐squared displacement plot at temperatures around and below the measured melting temperature of the LS12 hydrogel (ca. 58°C).

**Figure 3:**
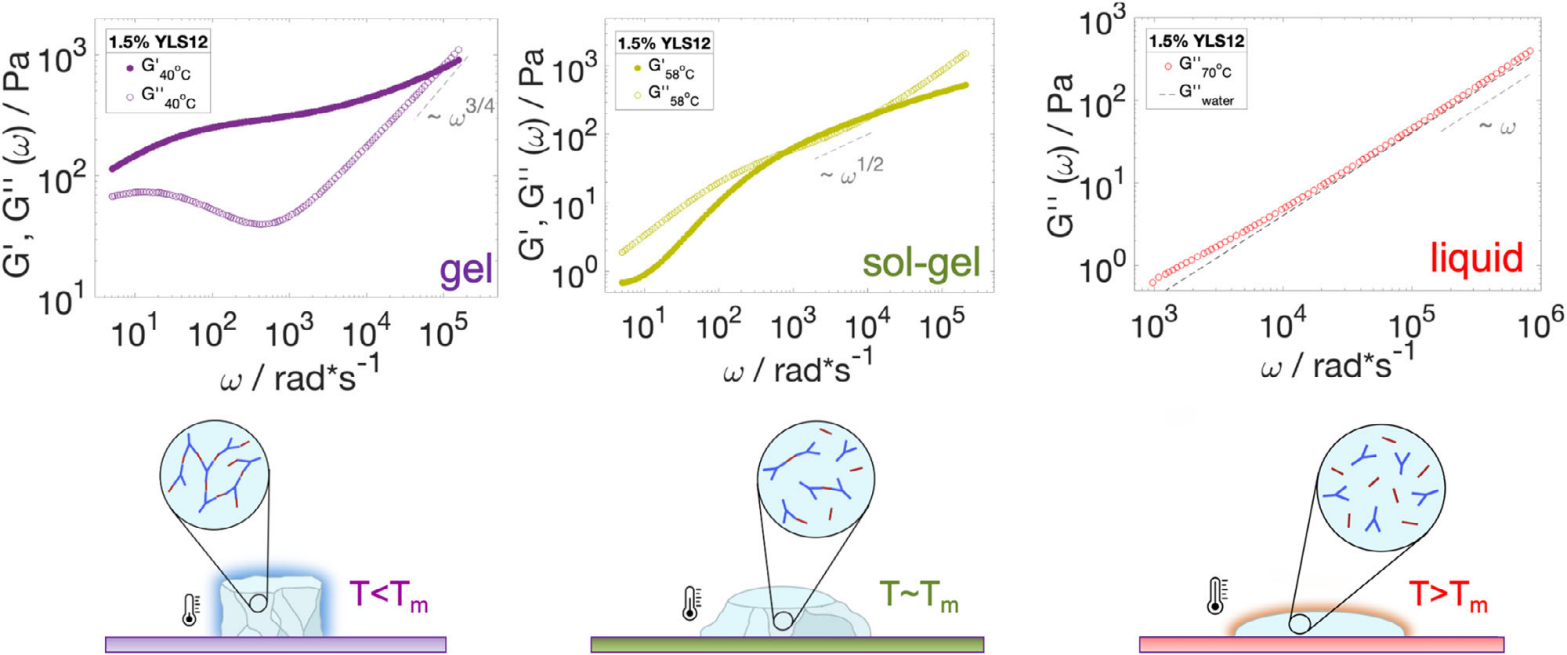
Fourier transforms allow conversion of the particle dynamics in Figure 2 into elastic, *G*′(ω) (filled symbols) and viscous, *G*″(ω) (empty symbols) moduli. At 70°C, all DNA strands dissociated and we found a viscosity similar to that of water (red data). At 40°C, YLS12 was found to be firmly in the gel state (violet data). The sol‐gel transition region for YLS12 was estimated to be around 58°C, where both moduli had a similar magnitude and nearly identical scaling of ω^0.5^ (green data). The DWS results are complemented with schematic depictions of the fully connected gel phase (violet, left), partially connected sol‐gel transition phase (green, middle) and totally dissociated liquid phase (red, right). The Newtonian linear scaling of *G*″(ω) at 70°C for the tracer particles in water is included as a reference (black dashed line).

To translate the tracer particle dynamics within LS12 into viscoelastic information about the hydrogel, we used the generalized Stokes–Einstein relation^[^ ^39^, ^40^ ^]^ and equivalence between Fourier and Laplace transforms to convert into the frequency‐dependent storage, *G*′(ω) and loss, *G*″(ω) moduli. These rheological parameters directly report on the solid‐like and liquid‐like properties of the material. **Figure** **3** illustrates the three characteristic regions within the viscoelastic spectrum of LS12. At temperatures below the melting temperature (*T* < *T*_*m*_), the storage modulus dominated over the loss modulus over a wide frequency window (10–100 000 rad · s^−1^). In the high‐frequency regime, the loss modulus first displayed a characteristic frequency scaling of ω^5/9^ for ω > 1000 rad · s^−1^ (Rouse–Zimm polymer behavior), and then ω^3/4^, where internal bending modes of Kuhn segments started to dominate. This change in exponent is commonly found in semi‐flexible polymer networks and occurs around a critical frequency ω_0_ that corresponds to the shortest relaxation time in the Rouse‐Zimm spectrum. With ω_0_ ≈ 11000 rad · s^−1^ and using the relation $\omega_{0}=k_{B}T/\left(8\eta_{s}l_{p}^{3}\right)$, where *k*_*B*_ *T* is the thermal energy and η_*s*_ ‐ the solvent viscosity, we directly deduced a persistence length *l*_*p*_ of around 42 nm (equivalent to about 130 base pairs).^[^ ^8^, ^41^, ^42^, ^43^, ^44^, ^45^ ^]^ At temperatures around the melting temperature (*T* ≈ *T*_*m*_), the approach of the gel point and onset of percolation was evident by the storage and loss moduli displaying similar magnitudes and identical frequency scaling of ω^0.5^ over a broad frequency range.^[^ ^46^ ^]^ At very high temperatures (*T* > *T*_*m*_), we measured negligible storage modulus and *G*″(ω) ∝ ω over a wide frequency window.

**Figure 4:**
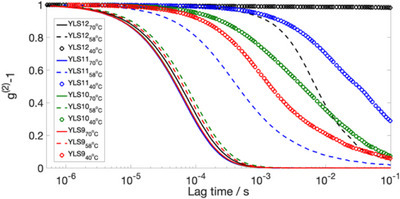
Passive microrheology using diffusing wave spectroscopy on 1.5 wt% YLS12, YLS11, YLS10, and YLS9 DNA hydrogels. To facilitate comparison between all hydrogels, we plot the raw intensity autocorrelation functions g^(2)^‐1 at fixed temperatures of 70°C (fully dissociated phase in all cases), 58°C (close to the sol‐gel transition temperature of YLS12) and 40°C (deep into the gel phase of YLS12). Comparison of the autocorrelation functions across all hydrogels shows that the curves decay more rapidly for shorter linkers. This decrease in decorrelation times from YLS12 to YLS9 implies a shift in melting temperature.

On replacing linkers LS12 with LS11, LS10, and LS9, we found an analogous thermally activated sol‐gel transition, with a decrease in the melting temperature of the DNA hydrogel upon deletion of bases from the sticky ends of the linkers (*cf*. **Figure** **4**). By synthetically mimicking the effect of introducing point mutations of type deletion, we progressively weakened the resulting hydrogel structure, where bases in the sticky ends of the Y‐shapes remained without hybridization partners. This decrease in enthalpic gain and increase in entropy could explain the shift of the sol‐gel transition to lower temperatures.

**Figure 5:**
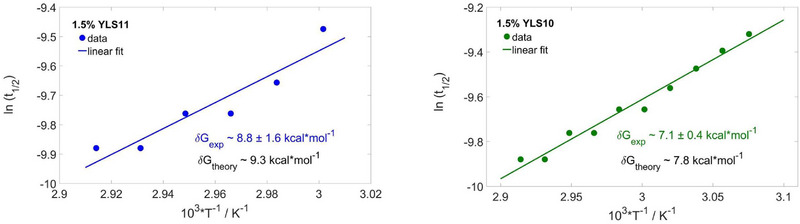
Arrhenius kinetics in 1.5 wt% YLS11 and YLS10 DNA hydrogels, taking into consideration the half‐decay time points of the intensity autocorrelation functions above the sol‐gel transition, where we expected a thermally activated network formation. We compare the activation free energy given by the extracted slopes from the logarithmic plots with the theoretical estimates by the nearest‐neighbor model of Allawi and Santalucia. We find reasonably good agreement (1–2 standard deviations) for systems bonded by sufficiently long sticky ends.

As the hydrogel formation is thermally activated following Arrhenius‐type kinetics, the half‐decay times of *g* ^(2)^ − 1 obey $t_{1/2}=t_{0}e^{-\delta G_{exp}/\left(RT\right)}$ and we can calculate the experimental activation energy δ*G*_*exp*_ at temperatures *T* > *T*_*m*_, where *R* represents the universal gas constant. **Figure** **5** displays the extracted energy activation barriers δ*G* for the 1.5 wt% YLS11 and YLS10 systems. In agreement with previous reports and the nearest‐neighbor model of Allawi and Santalucia,^[^ ^25^, ^47^, ^48^, ^49^ ^]^ we found lower energetic barrier for association‐dissociation kinetics with shorter sticky ends. We found a very close agreement between DWS‐based microrheology values for the activation barrier and the nearest‐neighbor model (Figure 5), with notable deviations only at shorter sticky‐end lengths, where the experimental activation barrier exhibited a steeper decline compared to theory (*cf*.  Figure S5, Supporting Information).

### 2.2 Bulk Rheology to Determine Sol‐Gel Transitions in Mixed‐Valency DNA Hydrogels

**Figure 6:**
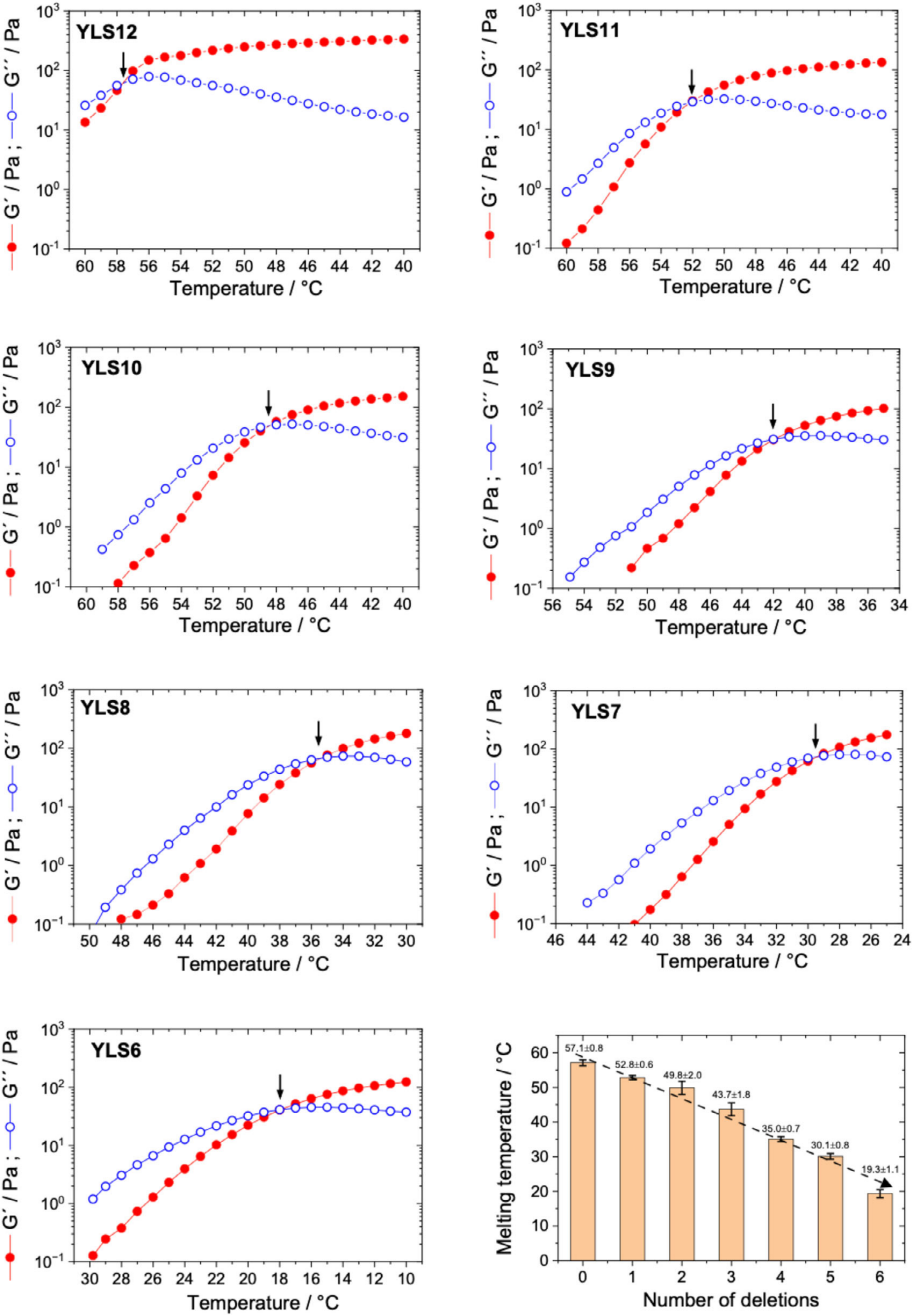
Temperature ramps in oscillatory bulk rheology on 1.5 wt% DNA hydrogels with linkers of different sticky‐end length. All ramps were performed at a fixed strain amplitude of 1% and frequency of 1 Hz. Red filled circles mark the elastic moduli *G*′(ω), while blue empty circles denote the loss moduli, *G*″(ω). The sol‐gel transition is indicated by an arrow in each case. The results were confirmed by performing multiple ramps at identical experimental conditions. The summary plot indicates how the melting temperature of the system changes upon removing bases from the sticky ends of the linkers. We found an approximately linear trend, where the melting temperature reduces by ca. 6°C per deleted base.

To cross‐validate and complement the passive microrheology results, we performed a bulk rheological study on all DNA‐hydrogel systems, from YLS12 to YLS6. The DWS data provided clear indication that removing DNA bases from the sticky ends of the linkers leads to a decrease in the sol‐gel transition temperature of the DNA hydrogel and reduction in the magnitudes of the storage and loss moduli measured at a fixed temperature. To confirm that trend, we plot in **Figure** **6** the individual cooling temperature ramps performed at 1 Hz oscillatory frequency and 1% strain, where we tracked the storage and loss moduli of each hydrogel over a temperature window of 20°C at a cooling rate of 0.005°C · s^−1^. In all samples, we found an initial dominance of the loss modulus over the elastic modulus at temperatures *T* > *T*_*m*_, where the melting temperature was identified as the point at which *G*′(ω) ≈ *G*″(ω). Past that point and at *T* < *T*_*m*_, the elastic modulus prevailed over the loss modulus and the material visibly turned into a percolating gel. By probing DNA hydrogels of concentration 1.5 wt%, we ensured a continuous sol‐gel transition and no phase separation into distinct DNA‐poor and DNA‐rich regions —a phase behavior previously observed in similar limited‐valency DNA‐nanostar mixtures of lower concentration.^[^ ^7^, ^50^, ^51^ ^]^ Notably, the bulk rheology data in Figure 6 confirmed that deleting bases from the sticky ends of the linkers shifts the sol‐gel transition to significantly lower temperatures — a decrease of 6°C per deletion on average. Obtaining similar quantitative structure‐property relations are anticipated to play a pivotal role in the model‐predictive design of DNA materials with tailored mechanical properties.

**Figure 7:**
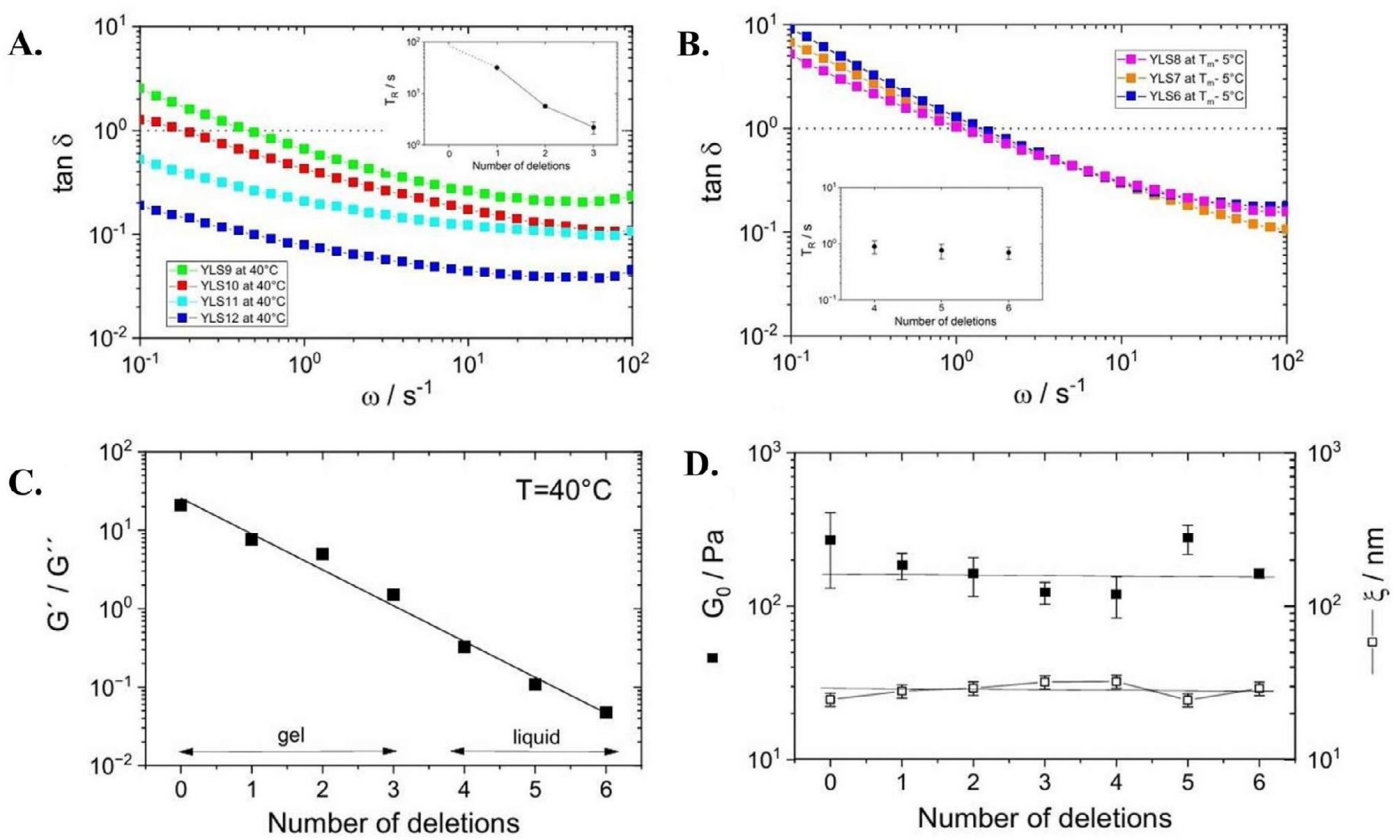
Extracted A,B) phase angles, C) relative stiffness, D) plateau elastic modulus and average mesh size in oscillatory bulk rheology on DNA hydrogels with linkers of different sticky‐end length. The phase angles in A) were extracted from frequency ramps at 40°C for YLS12, YLS11, YLS10 and YLS9, and in B) at 5°C below the corresponding melting temperature (*T* ≈ *T*_*m*_ − 5°C) of YLS8, YLS7, and YLS6. The strain in each case was fixed to 1%. A loss tangent of 1 marks the approximate transition point, where *G*′(ω) ≈ *G*″(ω). The insets in A) and B) show the relaxation times extracted from the corresponding frequency sweeps (*cf*. Figure S8, Supporting Information). All hydrogels were found to exhibit shear‐thinning behavior, with a reduction in viscosity on increasing frequency. C) The relative stiffness, defined as the ratio of *G*′(ω) and *G*″(ω), plotted for all hydrogels at a temperature of 40°C, strain of 1% and frequency of 1 Hz. We found an approximately linear decrease in relative stiffness with increasing the number of deleted bases. D) Approximately constant values for the plateau of the elastic modulus (filled squares) and associated average mesh size (empty squares) at low temperatures (*T* < *T*_*m*_) and fixed frequency of 10 Hz. The plateau values of the elastic modulus were obtained at temperatures differently spaced from the respective sol‐gel transitions, accounting for the large variations.

Moreover, to complete the mechanical characterization and enable further uses as biosensors, we probed via bulk rheology the frequency‐dependent relative stiffness of each hydrogel as measured by the phase angle δ = arctan(*G*″(ω)/*G*′(ω)) in the gel state. We split our DNA‐hydrogel systems into two categories: high‐temperature hydrogels containing target linkers, i.e. 1.5 wt% YLS12, YLS11, YLS10, and YLS9 measured at 40°C (**Figure** **7**A); low‐temperature hydrogels containing toehold linkers, i.e. 1.5 wt% YLS8, YLS7, and YLS6 measured at 5°C below the corresponding melting temperature (*T* ≈ *T*_*m*_ − 5°C) of each hydrogel (Figure 7B). Phase‐angle values of δ > 1 or δ < 1 are suggestive of predominantly viscous or elastic behavior, respectively.

At 40°C, we found that YLS12 and YLS11 exhibit gel‐like characteristics over a wide frequency range (0.1–100 rad · s^−1^) ‐ a behavior that extends to even higher frequencies, as confirmed by DWS microrheology. On further increasing the number of deletions, we observed that the transition from a terminal flow response to an elastic plateau regime shifts to higher frequencies (*cf*. inset of Figure 7A). This observation is consistent with a less stiff network that relaxes on a shorter timescale when subjected to an external shear stress. In contrast, we did not find a significant difference in the relaxation time on comparing YLS8, YLS7, and YLS6 at equidistant points from their respective sol‐gel transitions. This observation likely holds true for all hydrogel systems investigated here and confirms that reducing the number of DNA bases in the sticky ends of the linkers requires lower temperatures to obtain a mechanically equivalent response from the selected set of DNA hydrogels.

Further, at a fixed frequency of 1 Hz and temperature of 40°C, we observed a monotonic decrease in *G*′(ω)/*G*″(ω) upon removing more bases from the sticky ends of the linkers (Figure 7C). This decrease in relative stiffness is concomitant with shorter relaxation times of the network, where the highly interconnected three‐dimensional DNA hydrogel is capable of relaxing stresses more rapidly due to the incomplete binding between its building blocks, leaving unpaired bases on the Y‐shape arms. By performing frequency ramps in the gel state of all DNA hydrogels (Figure S8, Supporting Information), we found at 10 Hz an approximately constant elastic modulus plateau *G*_0_ of 120‐280 Pa (subject to large variation) and corresponding average network mesh size ξ ≈ $\sqrt[{3}]{k_{B}T/G_{0}}$ = 24‐32 nm (Figure 7D), as determined from classical theory of rubber elasticity.^[^ ^52^ ^]^ Moreover, the homogeneity of the hydrogels and their approximate pore size were confirmed by multi‐particle tracking (MPT, Figure S10, Supporting Information).

### 2.3 Linker Exchange for Biosensing Enabled by Complete Mechanical Characterization

Following a thorough initial characterization, we leveraged the mechanical tunability of our system to conduct linker‐exchange experiments. These were enabled by a toehold strand displacement mechanism which allows more thermodynamically favored DNA strands to use their enthalpic advantage in binding to target DNA strands and replace migratory strands that bind less favorably.^[^ ^53^, ^54^, ^55^ ^]^ This intricate mechanism has been previously used for the design of re‐entrant DNA gels and suggested as a powerful tool for discriminating single‐nucleotide variants (SNVs).^[^ ^56^, ^57^ ^]^

**Figure 8:**
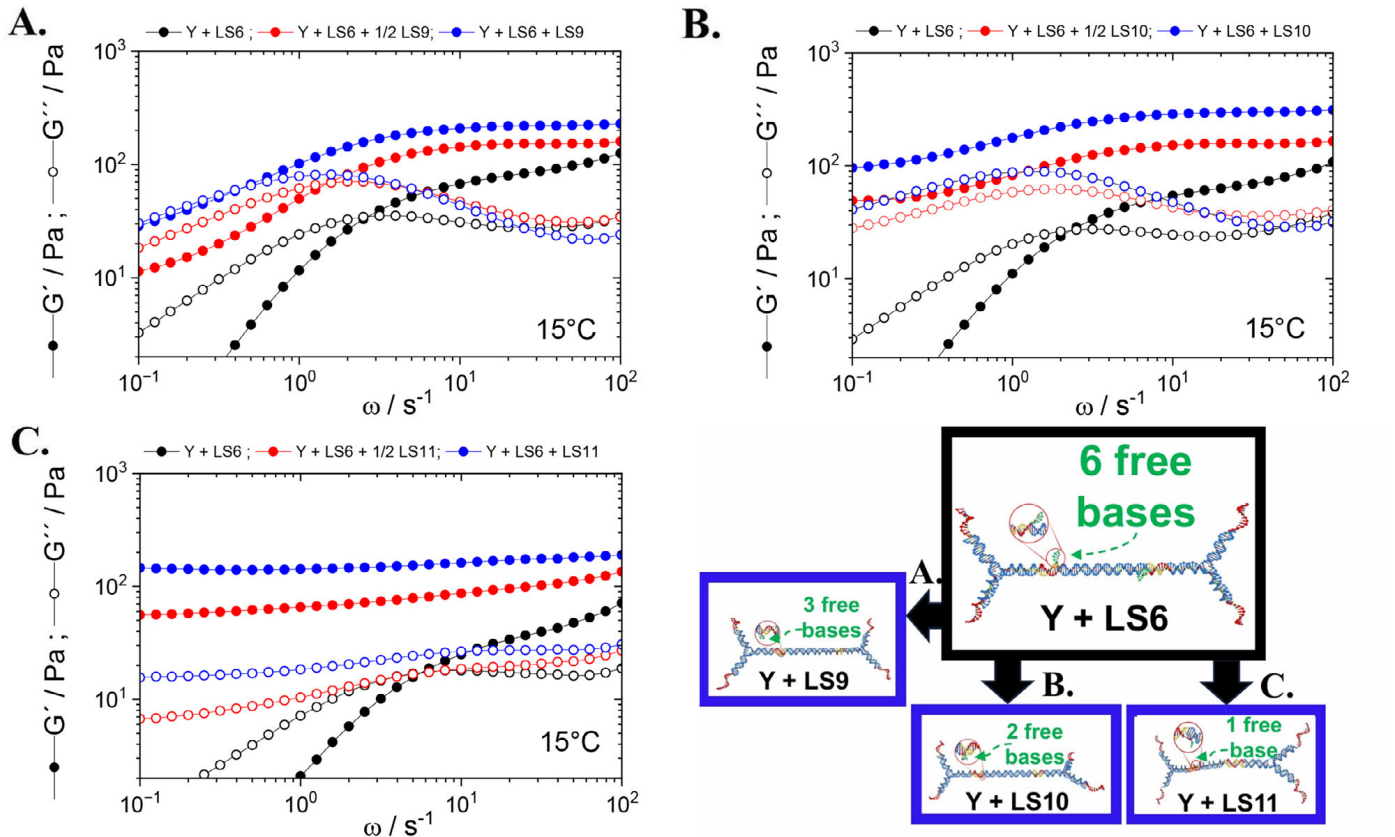
Linker‐exchange experiments: frequency sweeps in oscillatory bulk rheology at 15°C on 1.5 wt% YLS6 DNA hydrogels with Y:LS6 ratio of 2:3, ensuring that approximately all Y‐shapes are connected to LS6 toehold linkers. Adding A) LS9, B) LS10, or C) LS11 target linkers was then found to cause an increase in *G*′(ω) and *G*″(ω) in both concentration‐dependent and linker‐specific manner. Filled symbols signify the elastic modulus, *G*′(ω), whereas empty symbols mark the viscous modulus, *G*″(ω). Black symbols represent starting configurations (YLS6 hydrogels), red symbols mark the addition of target LS9, LS10, and LS11 at half the concentration of LS6 toehold linkers, and blue symbols denote further addition of target sequences, such that we obtain equal concentrations of toehold and target. We also show oxView schematic representations of the linker‐exchange process, where LS6 is replaced by LS9, LS10, or LS11 target linkers, respectively.

To test if our DNA‐hydrogel systems are capable of detecting and discriminating between SNVs, we performed further bulk rheology experiments (**Figure** **8**). Building on the measured difference in sol‐gel transitions, we started with the lowest temperature YLS6 DNA hydrogel containing LS6 toehold linkers. In separate experiments, we then added increasing amount of target A) LS9 linkers, B) LS10 linkers, and C) LS11 linkers, relying on the difference in binding energy with the Y‐shapes to trigger the exchange. The experiments were conducted at a fixed temperature of 15°C, thus working in the immediate proximity and below the sol‐gel transition of YLS6 (*T*_*m*_ − 5°C). The ratio of Y‐shapes to linkers in the YLS6 hydrogel was 2:3, ensuring only very few Y‐shape arms were free and available for further binding. Under this condition, any subsequent increase in viscoelasticity upon adding target linkers could be attributed to linker exchange. Here we note that adding inert linkers to an already formed hydrogel would have an effect of diluting the connected porous network, causing a decrease in *G*′(ω) and *G*″(ω). Owing to the rational design of our linkers, we found an increase in both moduli upon additions of LS9 (Figure 8A), LS10 (Figure 8B), and LS11 (Figure 8C). This boost in viscoelasticity was not observed upon addition of more LS6 linkers due to nanostar‐linker bond saturation, as expected.

**Figure 9:**
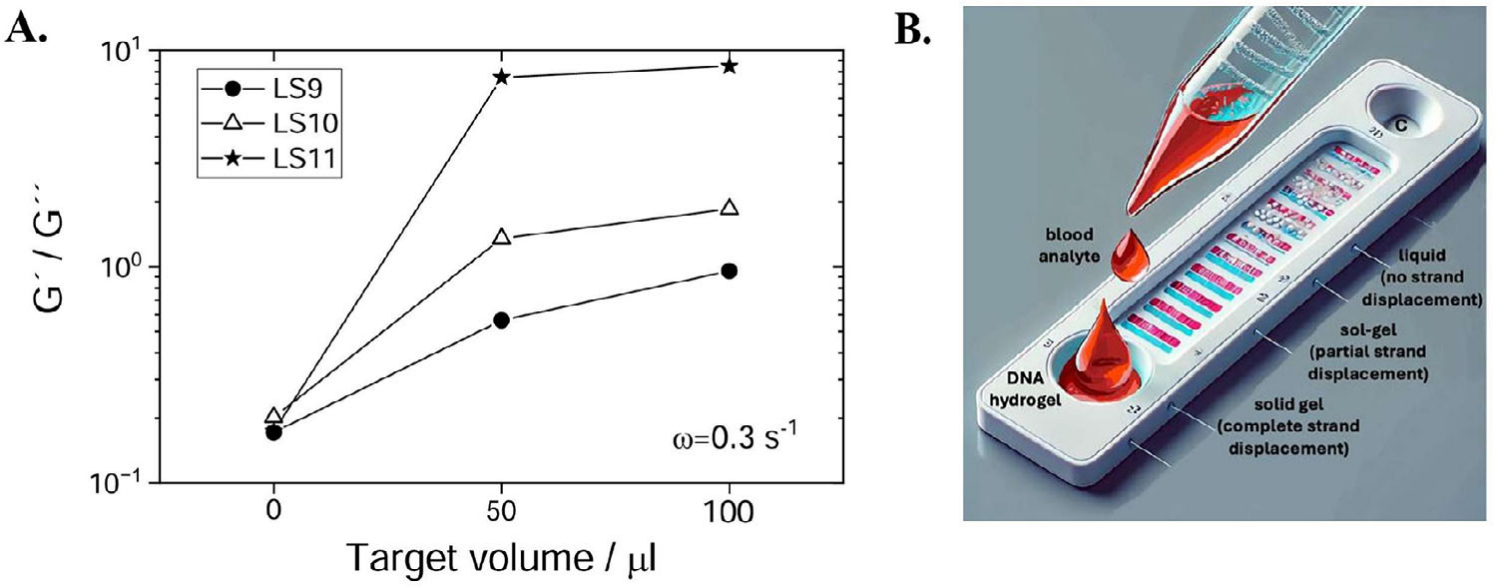
A) Increase in relative stiffness, *G*′(ω)/*G*″(ω) upon addition of LS9, LS10 and LS11 target linkers to YLS6 toehold hydrogel. The measurements were conducted at 10°C, 1% strain, and angular frequency of 0.3 s^−1^. Filled circles represent the volume of added LS9 linker, empty triangles ‐ LS10 linker, and filled stars ‐ LS11 linker. The differential increase in relative stiffness allows discrimination between targets, where the method appears to be more sensitive at lower volumes (< 50 µL) due to the counteracting dilution effect. B) A schematic representation of an example use case utilising a lateral flow test for the real‐time detection of a target gene within a few drops of a liquid (e.g. blood) sample. The lateral flow image was generated with the help of a language model (Dall‐E 2, OpenAI) and subsequently annotated.

In particular, our results suggest that the extent of increase in *G*′(ω) and *G*″(ω) upon addition of target sequences is highly dependent on the concentration and type of linker. For instance, adding low volume (concentration) of LS9, LS10, or LS11 to an already‐formed YLS6 triggered a more moderate increase in the moduli than doubling the volume (concentration) of the target. Similarly, adding LS10 or LS11 to YLS6 hydrogel caused a stronger increase in the elastic and viscous moduli compared to adding LS9, accompanied by an increase in the relaxation time of the network. Further, due to the counteracting effect of dilution, our toehold hydrogel system appeared to have stronger dose sensitivity to detecting targets of low volume (**Figure** **9**A). This observation suggests the high potential and promising uses of this mechanical sensing method in lateral flow assays, enabling rapid (nearly real‐time) detection of target genes (Figure 9B). Furthermore, the method is thermally reversible and can be used for multiple, sequential detections steps (Figure S7, Supporting Information).

We complement these findings with more examples of mechanical biosensing in Supporting Information (Figure S9), where we show alternative detection strategies that utilise lower concentrations of DNA and a different nanostar‐to‐linker ratio. For instance, we expect that nanostar‐to‐linker ratio of 1:1 leaves free Y‐shapes available for further binding, where even under minute differences in binding energy, one could for instance discriminate between detection of LS11 and LS12 of the same volume and concentration. On the other hand, operating close to the sol‐gel transition of, e.g., YLS8 could facilitate an amplified differential response in viscoelasticity upon addition of LS9, considering the significant difference in melting temperatures. Taken together, these observations suggest that, following a preliminary mechanical characterization, mixed‐valency DNA hydrogels could be used in rapid, nearly real‐time detection of point mutations in genetic disorders or for the identification of bacterial genomes in a diverse pool of other SNVs.^[^ ^58^, ^59^, ^60^ ^]^

## 3 Conclusion

Here we presented the design and mechanical characterization of a highly modular DNA system, *viz*. a DNA hydrogel composed of trivalent, branched DNA nanostars and bivalent, linear DNA linkers. A particular focus of this work is on the quantification of the relationship between the viscoelastic properties of a three‐dimensional, porous DNA network and the sequence composition of its constituting DNA oligomers. To this end, we determined through microrheology and bulk rheology that deleting bases from the sticky ends of the linkers (introducing synthetic point mutations) causes a drop of roughly 6°C in the sol‐gel transition temperature and lowers the magnitude of the storage and loss moduli under fixed experimental conditions (strain, temperature etc.). We then demonstrated how this high mechanical tunability could be utilized for biosensing applications involving linker exchange, where one could discriminate both for the concentration and type of detected linker. As an outlook extending from the results in this work, we envisage the design of real‐time alternatives to sequencing, where the presence of a bacterial genome or other pathogen is detected in situ through fine mechanical changes, leading to the execution of an action by a microscale mechanical actuator.

## 4 Experimental Section

### DNA Hydrogel Compositions and Synthesis

The DNA oligonucleotides in **Table** **1** were ordered from biomers.net GmbH, at synthesis scale of 10 µmol and HPLC‐purified. The oligonucleotides were initially freeze‐dried and then dissolved in 10 mm  phosphate buffer saline (PBS) supplemented with 100 mm  NaCl (pH 7.6). All oligo concentrations were then measured using NanoDrop 1000 and the hybridization behavior verified using polyacrylamide gel electrophoresis (PAGE) and UV‐visible (UV‐vis.) spectroscopy. Y1, Y2, and Y3 were designed to form a Y‐shape (trivalent nanostar), where linker strands LS‐1 and LS‐2 each formed a pair of bivalent DNA linkers. LS12 contained 12 bases in the sticky end (marked in red in Table 1), LS11 ‐ 11 bases in the sticky end, LS10 ‐ 10 bases in the sticky end etc. Y‐shape and linker strands were then separately subjected to a gradual polymerisation chain reaction (PCR) hybridisation protocol, heating from 25°C to 95°C followed by cooling from 95°C to 25°C over 9 h using a Doppio VWR thermocycler to ensure complete and thermodynamically stable hybridization. Due to losses in high‐performance liquid chromatography (HPLC) purification, the 10 µmol synthesis scale allowed us to obtain 1 mL of 600 µm stock linker and 1 mL of 400 µm stock Y‐shape, which we subsequently mixed in equal volumes to a final DNA concentration of 1.5 wt% (ca. 500 µm). This ensured maximum connectivity between the building blocks at an optimal ratio of Y:L = 2:3, compensating for the difference in valency.^[^ ^6^ ^]^ All samples were stored at 4°C and equilibrated to room temperature prior to spectroscopic and rheological experiments.

**Table 1:** DNA strands comprising Y‐shapes and linkers, with labeled sticky ends (red), Y1‐Y2 hybridizing segments (black), Y1‐Y3 hybridizing segments (olive), Y2‐Y3 hybridizing segments (violet), LS‐1 and LS‐2 hybridizing segments (blue). All synthesized DNA strands were purified via HPLC before use and hybridized into DNA building blocks via polymerase chain reaction (PCR).

DNA Strand Name	DNA Sequence
Y1	5' ‐ CGA TTG ACT CTC CAC GCT GTC CTA ACC ATG ACC GTC GAA G ‐ 3'
Y2	5' ‐ CGA TTG ACT CTC CTT CGA CGG TCA TGT ACT AGA TCA GAG G ‐ 3'
Y3	5' ‐ CGA TTG ACT CTC CCT CTG ATC TAG TA G TTA GGA CAG CGT G ‐ 3'
LS12‐1	5' ‐ GAG AGT CAA TCG TCT ATT CGC ATG ACA TTC ACC GTA AG ‐ 3'
LS12‐2	5' ‐ GAG AGT CAA TCG CTT ACG GTG AAT GTC ATG CGA ATA GA ‐ 3'
LS11‐1	5' ‐ GAG AGT CAA TC TCT ATT CGC ATG ACA TTC ACC GTA AG ‐ 3'
LS11‐2	5' ‐ GAG AGT CAA TC CTT ACG GTG AAT GTC ATG CGA ATA GA ‐ 3'
LS10‐1	5' ‐ GAG AGT CAA T TCT ATT CGC ATG ACA TTC ACC GTA AG ‐ 3'
LS10‐2	5' ‐ GAG AGT CAA T CTT ACG GTG AAT GTC ATG CGA ATA GA ‐ 3'
LS9‐1	5' ‐ GAG AGT CAA TCT ATT CGC ATG ACA TTC ACC GTA AG ‐ 3'
LS9‐2	5' ‐ GAG AGT CAA CTT ACG GTG AAT GTC ATG CGA ATA GA ‐ 3'
LS8‐1	5' ‐ GAG AGT CA TCT ATT CGC ATG ACA TTC ACC GTA AG ‐ 3'
LS8‐2	5' ‐ GAG AGT CA CTT ACG GTG AAT GTC ATG CGA ATA GA ‐ 3'
LS7‐1	5' ‐ GAG AGT C TCT ATT CGC ATG ACA TTC ACC GTA AG ‐ 3'
LS7‐2	5' ‐ GAG AGT C CTT ACG GTG AAT GTC ATG CGA ATA GA ‐ 3'
LS6‐1	5' ‐ GAG AGT TCT ATT CGC ATG ACA TTC ACC GTA AG ‐ 3'
LS6‐2	5' ‐ GAG AGT CTT ACG GTG AAT GTC ATG CGA ATA GA ‐ 3'

### Polyacrylamide Gel Electrophoresis (PAGE): Hybridisation Test for DNA Building Blocks

Polyacrylamide gel electrophoresis (PAGE) was conducted to verify the successful hybridization of DNA strands and to assess the purity of the oligonucleotide components used in the hydrogel assembly. Samples containing DNA nanostars, double‐stranded DNA linkers, and single oligonucleotides were individually loaded with a purple gel‐loading dye (6×, no SDS, B7025S, BioLabs, New England) onto a 10% non‐denaturing polyacrylamide gel prepared in 10× TBE buffer (Tris‐Borate‐EDTA) with 11 mm+  MgCl_2_. The gel was run at a constant voltage of 100 V for 1 h at room temperature. Following electrophoresis, the gel was stained with GelRed nucleic acid stain in water (3×, Biotium, 41001) for 30 min and visualized under UV illumination using a Gel Doc imaging system. The migration patterns of the DNA components were compared to a DNA ladder (Quick‐Load Purple 1kb Plus DNA Ladder, NO550S, BioLabs, New England) to confirm the formation of the intended DNA structures and to estimate their size. Bands corresponding to hybridized DNA nanostars and other assemblies were qualitatively inspected for intensity and sharpness (*cf*. Figure S1, Supporting Information).

### UV‐Visible Spectroscopy: Melting Behavior of DNA Building Blocks

UV‐visible absorption spectra (Figure S2, Supporting Information) were recorded at a wavelength of 260 nm by Cary 3500 Multicell Peltier UV‐visible spectrophotometer by Agilent. At this wavelength we made use of the differential absorbance of single‐stranded and double‐stranded DNA to confirm the formation of all Y‐shapes and linkers, as well as obtain information about their melting temperatures (*cf*. Tables S1 and S2, Supporting Information) under different solvent and heating rate conditions. All temperature ramps (heating and cooling) were performed between 25°C and 85°C at a controlled rate of 1°C · min^−1^ that minimized hysteretic effects. The oligos were dispersed in phosphate buffer saline (PBS comprised of 10 mm  PB and 100–200 mm  NaCl, pH ≈ 7.4) at concentrations close to 1 µm , ensuring absorbance values within the specified range of operation of Cary 3500. The melting temperature (*T*_*m*_) was determined as the mid‐point of the sigmoidal melting curve, where on average half of all hydrogen bonds were broken (heating) or formed (cooling). The uncertainties in the melting temperatures were quantified as the difference between the values measured in heating and cooling ramps.

### Diffusing Wave Spectroscopy (DWS): Sampling Rapid Dynamics of Network Assembly

Diffusing wave spectrometer (DWS RheoLab, LS Instruments) equipped with echo mode was used to probe in transmission geometry the passive microrheology of the DNA hydrogels (1.5 wt%, 100 µL per measurement) over long correlation times. The samples were loaded into quartz cuvettes with a path length of 1 mm and within the sample we embedded 260‐nm polystyrene tracer particles of 2 wt% concentration and surface‐coated with polyethylene glycol to provide steric stabilization. A coherent laser light source with 685 nm wavelength was used to illuminate the samples and the scattered light intensity was collected by a highly sensitive avalanche photodiode. The intensity autocorrelation function was analyzed to extract the mean‐squared displacement (MSD) of the tracer particles. The storage and loss moduli were calculated using the generalized Stokes–Einstein relation in Laplace space ($\tilde{G}\left(s\right)\approx \frac{k_{B}T}{\left(\pi Rs\tilde{\text{MSD}}\left(s\right)\right)}$), enabling the conversion from tracer particle dynamics (tracers of size R) to assessment of the gel's microstructural properties and dynamic behavior across a wide frequency range (Figures S3 and S4, Supporting Information).

### Bulk Rheology: Comparison of Mechanical States Across DNA‐Hydrogel Systems

The bulk rheological properties of the DNA hydrogels (1.5 wt%, 200 µL per measurement) were measured using a stress‐controlled rheometer (MCR Physica 501, Anton Paar) equipped with a cone‐and‐plate geometry (CP25, 25‐mm diameter, 1‐mm gap). Frequency sweeps were conducted over an angular frequency range of 0.1–100 rad · s^−1^ at a fixed strain amplitude of 1%, ensuring measurements within the linear viscoelastic region. Temperature sweeps were performed in the range 60°C to 10°C at a cooling rate of 0.005°C · s^−1^ to probe sol‐gel transitions. The elastic modulus, *G*′(ω) and loss modulus, *G*″(ω) were recorded as functions of frequency and temperature to characterize the viscoelastic response of the hydrogels. Multiple measurements were performed to quantify the uncertainty.

## Conflict of Interest

The authors declare no conflict of interest.

## Author Contributions

AEC, AWUA, and IDS performed the experiments and analyzed the DWS microrheology data. AE and AWUA performed all UV‐visible spectroscopy and gel electrophoresis experiments, confirming the intended design of the DNA oligos. IDS conceived the experiments and wrote the first draft of the manuscript. CO analyzed all bulk rheology data. NW read and critically evaluated the manuscript. All authors read and provided input to the final version.

## Supporting information

Supporting Information

## Data Availability

The data that support the findings of this study are available from the corresponding author upon reasonable request.
