# Supplementary material for: Mechanically Tunable DNA Hydrogels as Prospective Biosensing Modules
Source: Macromol Rapid Commun. 2025 May 8;46(23):2500149. doi: 10.1002/marc.202500149 (PMC12687723; doi:10.1002/marc.202500149)
Supplement: Supplementary file 1 — Supporting Information [file MARC-46-2500149-s001.pdf]

Supplementary Information

Asya E. Can,[1] Abdul W. U. Ali,[1] Claude Oelschlaeger,[2]
Norbert Willenbacher,[2] Iliya D. Stoev[1]*

[1]*Institute of Biological and Chemical Systems - Biological Information Processing, Karlsruhe Institute of Technology, Eggenstein-Leopoldshafen 76344, Germany*
[2]*Institute for Mechanical Process Engineering and Mechanics, Karlsruhe Institute of Technology, Karlsruhe 76131, Germany*
*Correspondence to be addressed to: iliya.stoev@kit.edu

1 Polyacrylamide gel electrophoresis (PAGE)

We used polyacrylamide gel electrophoresis (PAGE) to confirm the assembly of Y-shaped nanostars and double-stranded linear linkers used as building blocks in the subsequent DNA hydrogels (*cf.* Supplementary Figure S1). DNA strands Y1, Y2 and Y3 were designed to be partially complementary to each other, allowing the formation of trivalent DNA nanostars that can bind to a total of three linkers. The linkers were designed such that every LS-1 has a hybridising partner LS-2, to which it binds incompletely, allowing for the formation of a further bond with an arm of a Y-shape through complementary sticky ends. The electric field was applied such that DNA migrated from top to bottom (negative to positive electrode). The final composition of the gel matrix was 10 vol% polyacrylamide and 11 mM $MgCl_2$.

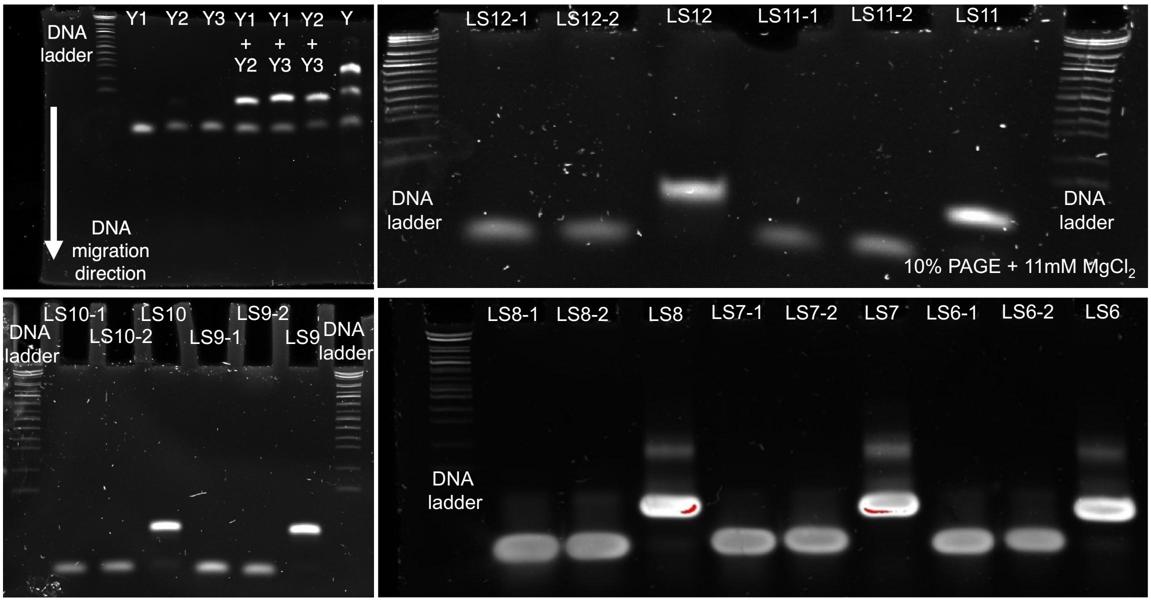

Supplementary Figure S 1: The migration profiles of all building blocks used for the formation of DNA hydrogels in this study and their respective intermediates (Y1, Y2, Y3, LS-1 and LS-2). The 2-log DNA ladders (100-10,000 bases) serve as molecular rulers and provide size and conformation reference. Clear and distinct bands confirm the successful formation and purity of the desired DNA assemblies, with migration direction indicated from top to bottom.

We attributed the presence of side bands to imperfect match in stoichiometry, leaving some Y-strands or L-strands without a partner. We expected minimal impurities due to performing HPLC purification following synthesis. Naturally, the bulkier and more highly branched trivalent Y-shapes diffused a shorter distance through the pores of the polyacrylamide gel matrix, whereas the smaller and approximately linear DNA linkers diffused further. In accordance with our expectations, the single-stranded DNA oligomers migrated the longest distance.

2 UV-visible spectroscopy

Supplementary Figure S2 summarises the melting temperatures of our building blocks, showing increase with salt concentration and lower hysteresis loop with lower temperature rates.

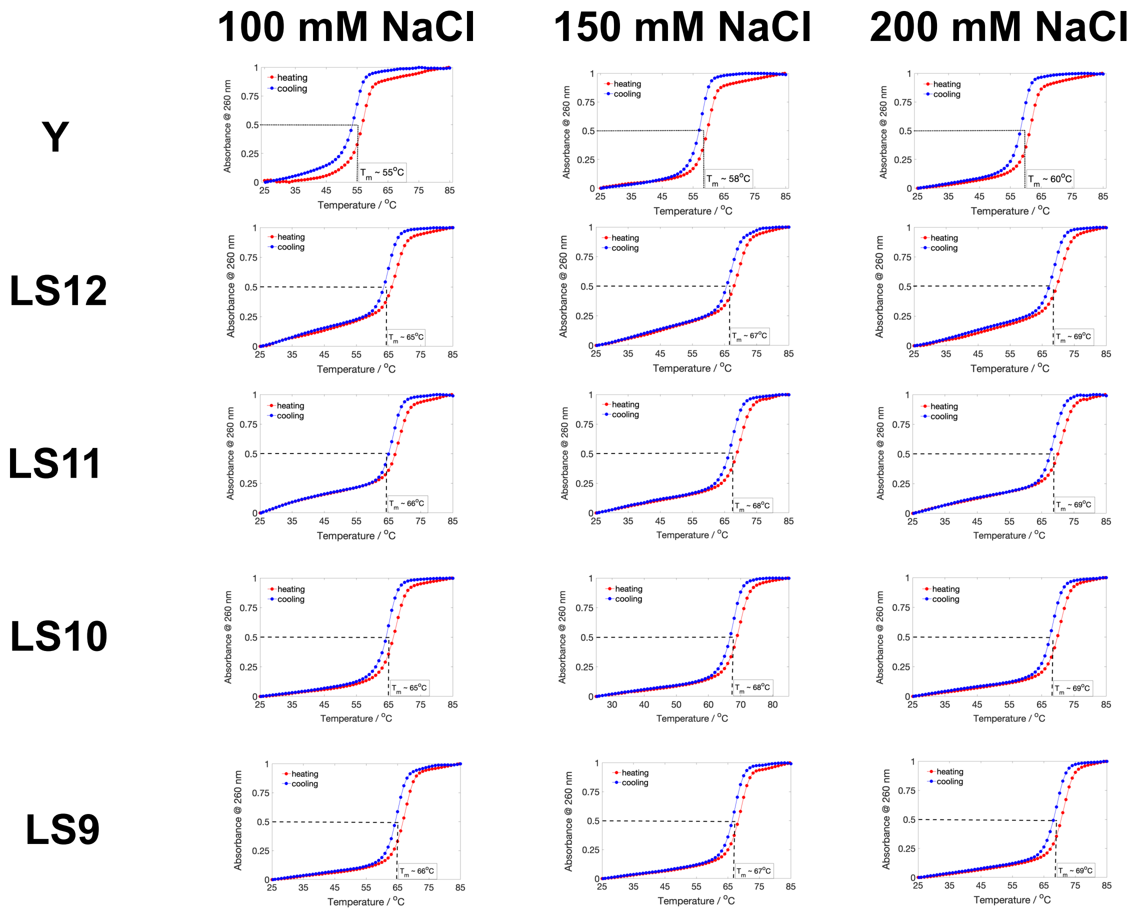

Supplementary Figure S 2: Melting curves of 1 μM DNA building blocks (Y-shapes and linkers) in 10 mM phosphate buffer saline (pH 7.4), obtained from UV-visible spectroscopy at different salt concentrations (100-200 mM) and temperature rate of $1°C \cdot min^{-1}$.

Supplementary Table S1 provides a summary of the melting temperatures (T_m) of Y-shaped DNA nanostructures at varying rates of temperature change and buffer compositions. The average melting temperatures obtained from heating and cooling ramps exhibited very little to negligible dependence on the precise rate of temperature change, with higher rates (e.g. $5°C \cdot min^{-1}$) naturally showing greater uncertainty in T_m estimation. Additionally, increasing the NaCl concentration from 100 mM to 200 mM resulted in a consistent increase in T_m, indi-

cating enhanced thermal stability due to the screened electrostatic repulsion between individual negatively charged DNA strands.

Supplementary Table S2 provides the melting temperatures of DNA assemblies, both Y-shaped nanostars and double-stranded DNA linkers, at a fixed rate of temperature change of $1°C \cdot min^{-1}$. We found that T_m varies very slightly and within measurement error across different linker compositions. Moreover, we expect that T_m increases with DNA concentration, so at hydrogel-relevant concentrations of hundreds of μM both Y-shapes and linkers would associate at temperatures higher than 60°C. This was taken into consideration when designing the sticky ends of the Y-shapes and linkers, thereby ensuring the step-by-step hierarchical assembly of DNA hydrogels, where the latter must necessarily have a melting temperature lower than that of the comprising building blocks. Therefore, in designing a DNA hydrogel, one strictly follows the trend: $T_{m,hydrogel} < T_{m,Y-shapes} \approx T_{m,linkers}$.

In the extracted experimental values below, we used low DNA concentrations (sub-micromolar), ensuring the validity of the assumptions underlying Beer-Lambert law. Further detailed studies into the kinetics of DNA-nanomotif assembly would be valuable in identifying the thermally driven formation of secondary structures and metastable configurations representing local minima in the potential energy landscape.

**Rate of Y-shape assembly** [$°C \cdot min^{-1}$]	T_m **in 10 mM PB 100 mM NaCl** [°C]	T_m **in 10 mM PB 150 mM NaCl** [°C]	T_m **in 10 mM PB 200 mM NaCl** [°C]
0.1	55.48 ± 1.10	58.32 ± 0.00	59.61 ± 0.11
0.5	56.09 ± 0.92	58.66 ± 0.78	59.57 ± 0.93
1	55.91 ± 1.17	58.25 ± 1.32	59.73 ± 1.53
2	56.08 ± 2.52	58.22 ± 2.38	59.77 ± 3.23
5	55.68 ± 5.89	58.55 ± 5.56	59.52 ± 6.01

Supplementary Table S 1: Y-shape melting temperatures under varying buffer compositions and rates of temperature change.

**DNA building block assembly**	T_m **in 10 mM PB 100 mM NaCl** [°C]	T_m **in 10 mM PB 150 mM NaCl** [°C]	T_m **in 10 mM PB 200 mM NaCl** [°C]
Y-shaped nanostar	55.07 ± 1.61	58.25 ± 1.32	59.73 ± 1.53
LS12 linker	64.77 ± 1.23	66.80 ± 1.14	68.33 ± 1.17
LS11 linker	66.05 ± 1.02	67.58 ± 1.24	68.81 ± 1.19
LS10 linker	65.39 ± 1.13	67.79 ± 1.06	68.72 ± 1.16
LS9 linker	65.51 ± 1.22	67.33 ± 0.96	69.28 ± 1.22

Supplementary Table S 2: Melting temperature analysis obtained from sigmoid-curve fitting at fixed temperate rate of $1°C \cdot min^{-1}$ and varying salt concentration.

3 Diffusing wave spectroscopy (DWS)

Diffusing wave spectroscopy (DWS) was employed to investigate on small scale the viscoelastic properties of the DNA hydrogels, enabling the characterisation of their dynamic sol-gel transition behaviour across a wide frequency range. In Supplementary Figure S3 we note that starting from 70°C and cooling to temperatures around 20-30°C, all samples display a transition from a liquid suspension into a gel, as indicated by the progressively longer decorrelation times. The delayed onset of gelation upon removing bases from the sticky end of the linkers can also be clearly seen. The signal-to-noise was found to decrease significantly upon entering deeply into the gel phase, where the system became progressively more and more non-ergodic.

We also converted the autocorrelation functions into mean-squared displacements (MSDs) that show how the tracer particles diffuse within each hydrogel at different temperatures (Supplementary Figure S4). The emergence of sub-diffusive region in the MSD signified the onset of gelation and corresponded to the approximate sol-gel transition, as determined also in bulk rheology. Following mode-coupling theory, each tracer particle performs cage hopping from one confined space within the gel onto another. Given a sufficiently high frequency of attempts, the particles eventually leave the local confines of the gel and are observed to sample the bulk liquid behaviour on the longest timescales probed here.

In the main manuscript we provided analysis of the Arrhenius kinetics of thermal activation for the formation of YLS11 and YLS10 hydrogels. The data were collected by extracting the half-decay points of the ACFs plotted in Supplementary Figure S3. On deleting bases from the sticky ends of the linkers, we effectively shortened the hybridising segments that connect those linkers to the Y-shapes. In Supplementary Figure S5 we point out that moving from 11 to 9 bases in the sticky end, we already find certain discrepancies in the experimentally determined activation energy and the one predicted by the nearest-neighbour model of Allawi and Santalucia. These differences could be attributed to the approximations underlying the model, which do not take into account long-range base interactions. However, determining experimentally the precise activation energy barrier could be important for biosensing applications, where differences in energetics are utilised for the replacement of toehold DNA strands with more thermodynamically favoured ones.

To this end, we performed DWS experiments to test if Y-shapes preferentially bind to linkers with longer complementary sticky ends (Supplementary Figure S6). We first prepared 1.5 wt% YLS6, which we measured at 60°C and 10°C, obtaining a significant difference in viscoelastic response, confirming the formation of a hydrogel network, as in Supplementary Figure S3. Then, at 60°C we added LS12 linkers at an equimolar amount to the LS6 already present in solution. Maintaining the same number of Y-shapes, this also had a diluting effect since about half of all linkers remained without an available binding site. Nevertheless, we found a significant increase in the decay time of the autocorrelation function upon adding LS12, confirming that Y-shapes preferentially bind to LS12 at the expense of LS6. This behaviour can be rationalised by the difference in binding energy and hints at the possibility of optically detecting the presence of target genomes in biosensing. We point out that as we performed this experiment at temperatures much higher than the melting transition of YLS6, this type of sensing differs from the linker exchange presented in the main manuscript, which perhaps remains the more robust detection method.

Finally, we performed DWS experiments to probe the capability of our DNA hydrogels to sense multiple targets and reversibly return to their starting mechanical state (Supplementary Figure

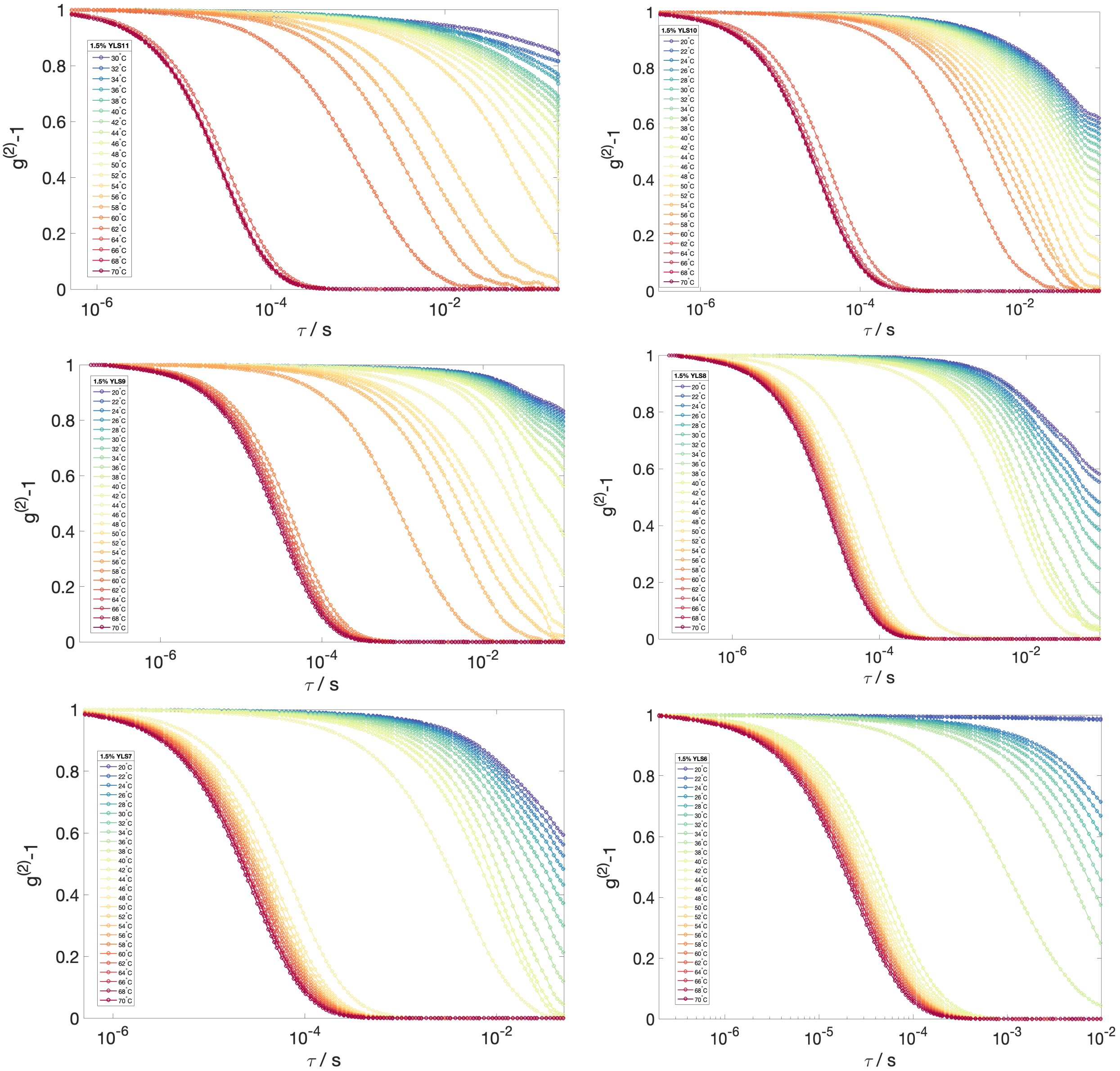

Supplementary Figure S 3: Intensity autocorrelation functions for all hydrogels besides YLS12 (1.5 wt% concentration), given as functions of lag time τ and temperature. The approach towards gelation can be clearly seen by the longer decorrelation times upon cooling, starting from 70°C and reaching $20 - 30$°C. We note slight discrepancies deep into the gel phase, where non-ergodicity no longer allows accurate translation of particle dynamics into the material's viscoelastic response.

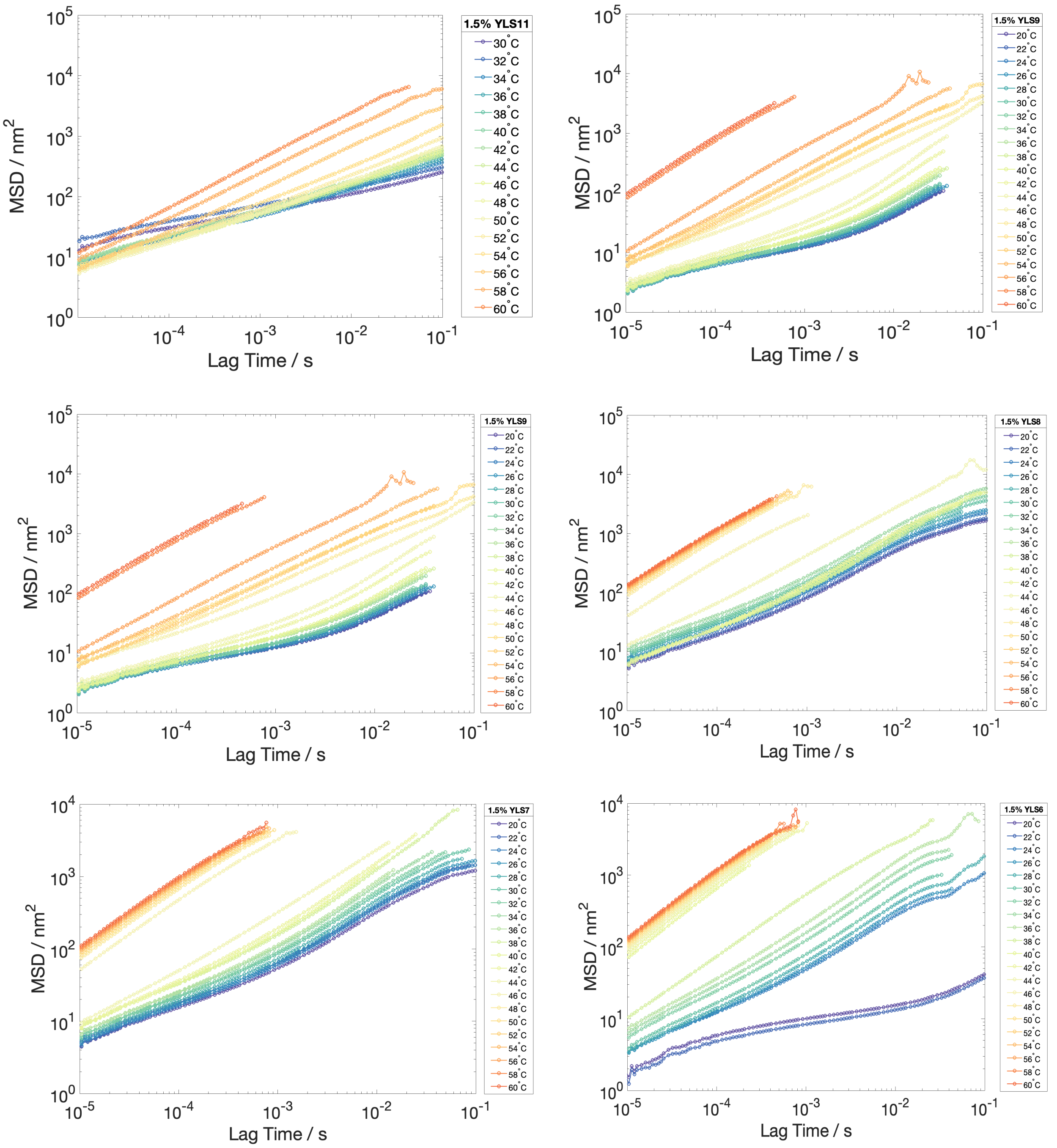

Supplementary Figure S 4: Mean-squared displacement functions for all hydrogels besides YLS12 (1.5 wt% concentration), given as functions of lag time and temperature. We plot the MSDs starting from the formation of the building blocks around 60°C and deep into the gel phase, *i.e.* 20 − 30°C. Despite challenges related to the accurate determination of the melting transition in the DWS experiments, we found an overall good agreement with macroscopic bulk rheology.

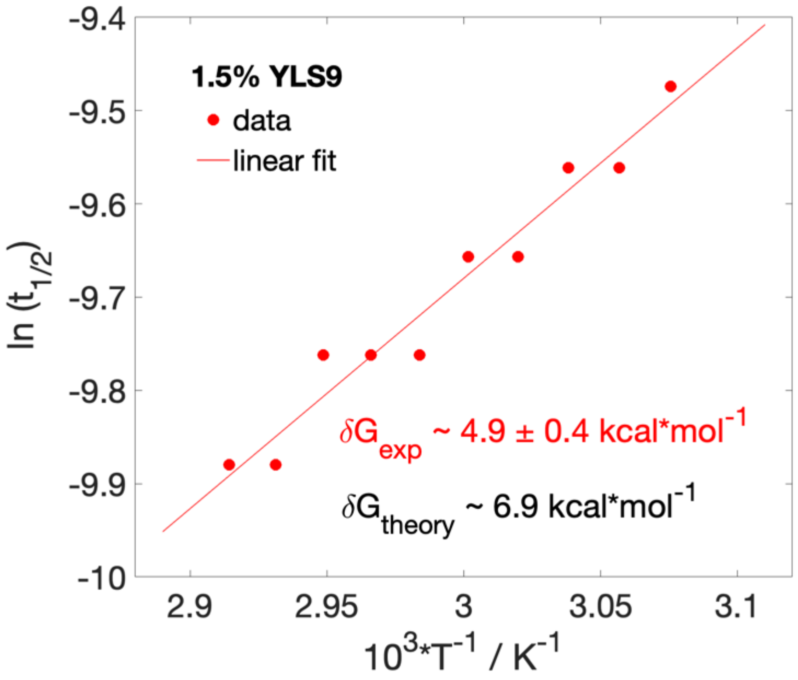

Supplementary Figure S 5: Arrhenius kinetics in 1.5 wt% YLS9 DNA hydrogel extracted from the half-decay time points of the intensity autocorrelation functions above the sol-gel transition, where we expected a thermally activated network formation. We compare the activation free energy given by the extracted slope to the theoretical estimate by the nearest-neighbour model of Allawi and Santalucia. There are notably higher deviations compared to comparing the same systems with longer sticky ends.

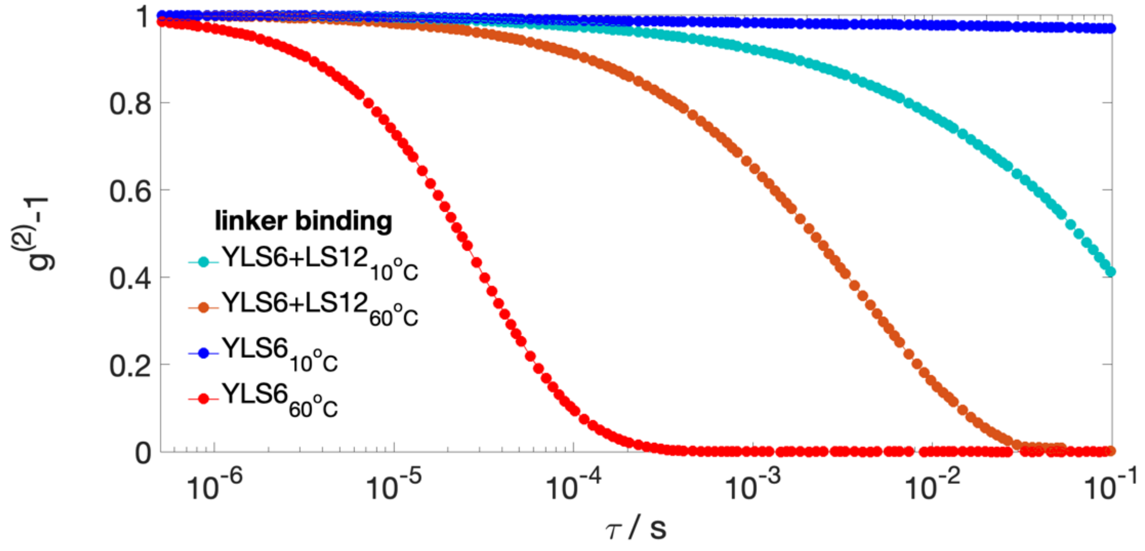

Supplementary Figure S 6: Preferential binding of Y-shapes to linkers LS12 over LS6, starting from 1.5 wt% YLS6 DNA hydrogel. In agreement with Supplementary Figure S3 we observe an increase in the correlation time on cooling for YLS6. However, adding LS12 linkers in an already established network of Y and LS6 leads to unbinding of the LS6 and binding of LS12, as is evidenced by the large shift in correlation time at 60°C.

S7). We first prepared 1.5 wt% YLS6, where we demonstrated how one returns to the same mechanical state using temperature as a switch. Starting with YLS6 at 60°C (red circles), we observed an increase in relaxation time due to progress towards gelation at 15°C (cyan diamonds). Then, we returned to 60°C (red crosses), where we observed the same relaxation as at the start. Next, upon addition of a target (here LS9) at 60°C (orange circles), we reliably and reversibly changed the mechanical response, first cooling to 35°C (blue-green diamonds) and then heating back to 60°C (orange crosses). Finally, we added a second target (here LS12) at 60°C (yellow circles) and observed a change to longer relaxation times at 10°C (blue diamonds), until returning back to 60°C (yellow crosses). To sum up, using the same sample we were able to detect two different targets in successive steps (despite diluting the initial hydrogel upon addition of targets) and we achieved complete reversibility each time.

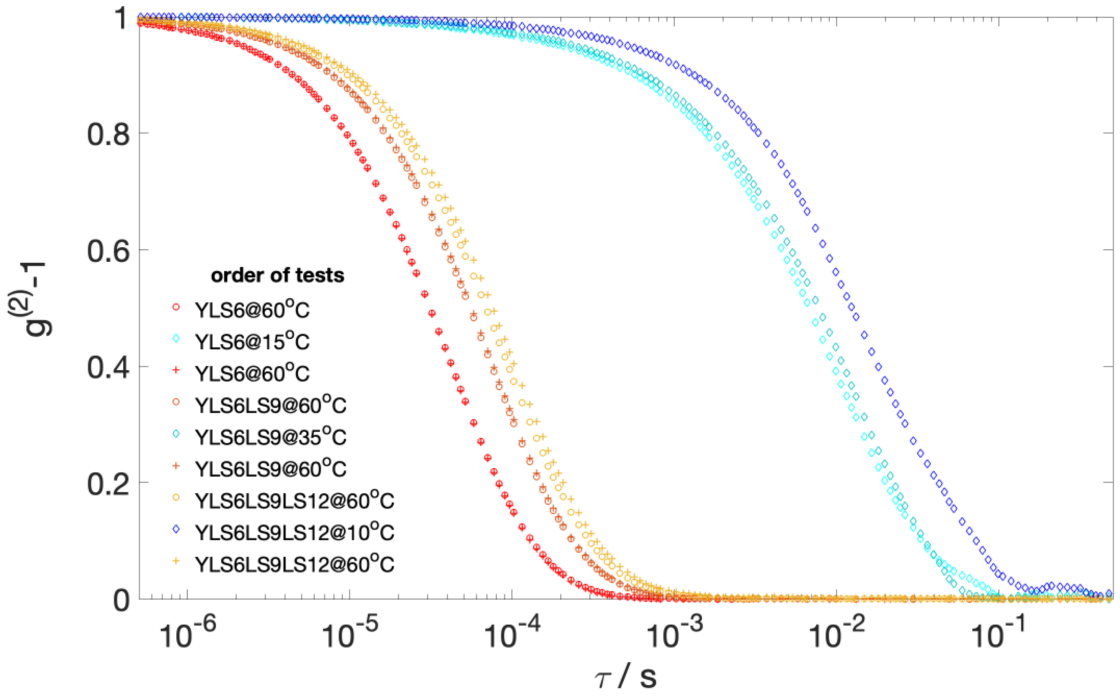

Supplementary Figure S 7: Intensity autocorrelation functions for: 1.5 wt% YLS6 hydrogel measured at 60°C (red circles), then 15°C (cyan diamonds), and again at 60°C (red crosses); YLS6 + LS9 target at 60°C (orange circles), then 15°C (blue-green diamonds), and again at 60°C (orange crosses); YLS6 + LS9 + LS12 targets at 60°C (yellow circles), then 15°C (blue diamonds), and again at 60°C (yellow crosses). This demonstrates the complete reversibility of the biosensing process and the multi-purpose, multi-use detection of targets.

4 Bulk rheology

In Supplementary Figure S8 we show the frequency sweeps obtained in measuring all 1.5 wt% hydrogels with a bulk rheometer in oscillatory downward ramps from 100 to 0.1 rad·s^{-1} at a fixed strain amplitude of 1%. As in Figure 7 of the main manuscript, we split the hydrogel systems into two categories, measuring high-temperature target hydrogels YLS12, YLS11, YLS10 and YLS9 at 40°C, and low-temperature toehold hydrogels YLS8, YLS7 and YLS6 at 5°C below their corresponding melting temperatures.

We note that YLS12, YLS11, YLS10 and YLS9 turn into gels at very different temperatures (spaced by an average of 6°C), explaining the modified appearance of the frequency ramps when measured at a constant temperature of 40°C. The relaxation time and absolute values of the storage and loss moduli of the gel network can thus be readily altered through linker exchange, opening up the possibility for detecting single-nucleotide variants in cascaded toehold strand displacement reactions. Similarly, selecting low-temperature toehold YLS8, YLS7 or YLS6 hydrogels close to their respective melting temperatures was expected to allow concentration and composition sensitive detection of the presence of known genomes, here corresponding to sequences YLS9, YLS10, YLS11 or YLS12.

Building on this idea, we used toehold strand displacement mechanism in our thoroughly mechanically characterised hydrogels to exemplify the detection of those target sequences. In Supplementary Figure S9 we show frequency sweep data from linker-exchange experiments at a fixed strain amplitude of 1%. In Supplementary Figures S9A-B, we measured at fixed temperatures of 25°C (ca. $T_m - 10$°C for YLS8) and 30°C (ca. $T_m - 5$°C for YLS8), respectively. Operating so close to the sol-gel transition of YLS8, we obtained an amplified viscoelastic response upon addition of LS9 at only half the original concentration of toehold linkers. To facilitate the detection, these experiments involving only one change in sticky-end base sequence were performed on 1 wt% DNA hydrogels. That linker exchange indeed took place is evident also from the increase in relaxation time, accompanying stronger binding of Y-shapes to LS9 target sequences.

Moreover, in Supplementary Figure S9C we began by measuring YLS9 DNA hydrogel at a concentration of 1 wt% at 10°C and followed the concentration-dependent viscoelastic response upon addition of LS12 target linkers. In spite of moderate sample dilution and working deep into the gel state of both systems, we observed a steady increase in both the elastic, $G'(\omega)$ and viscous, $G''(\omega)$ moduli, concomitant with an increase in LS12 concentration.

In Supplementary Figure S9D, we measured 1.5 wt% YLS11 hydrogel at 10°C using 1:1 ratio of Y-shapes and linkers. This way, even though we measured our system deep into the gel state, we were able to detect differential viscoelastic response on addition of LS11 or LS12 in two separate experiments performed under identical conditions. As expected, we observed ever so slightly higher increase in $G'(\omega)$ and $G''(\omega)$ on adding LS12 linkers. Taken together with the linker-exchange experiments in Figure 8 of the main manuscript, we showed how one could mechanically detect single-nucleotide changes at micromolar concentrations and discriminate between target sequences. This novel type of mechanical biosensing could provide an appealing alternative to sequencing methods, obviating the need for time-consuming analysis of long reads and allowing applications *in-situ*.

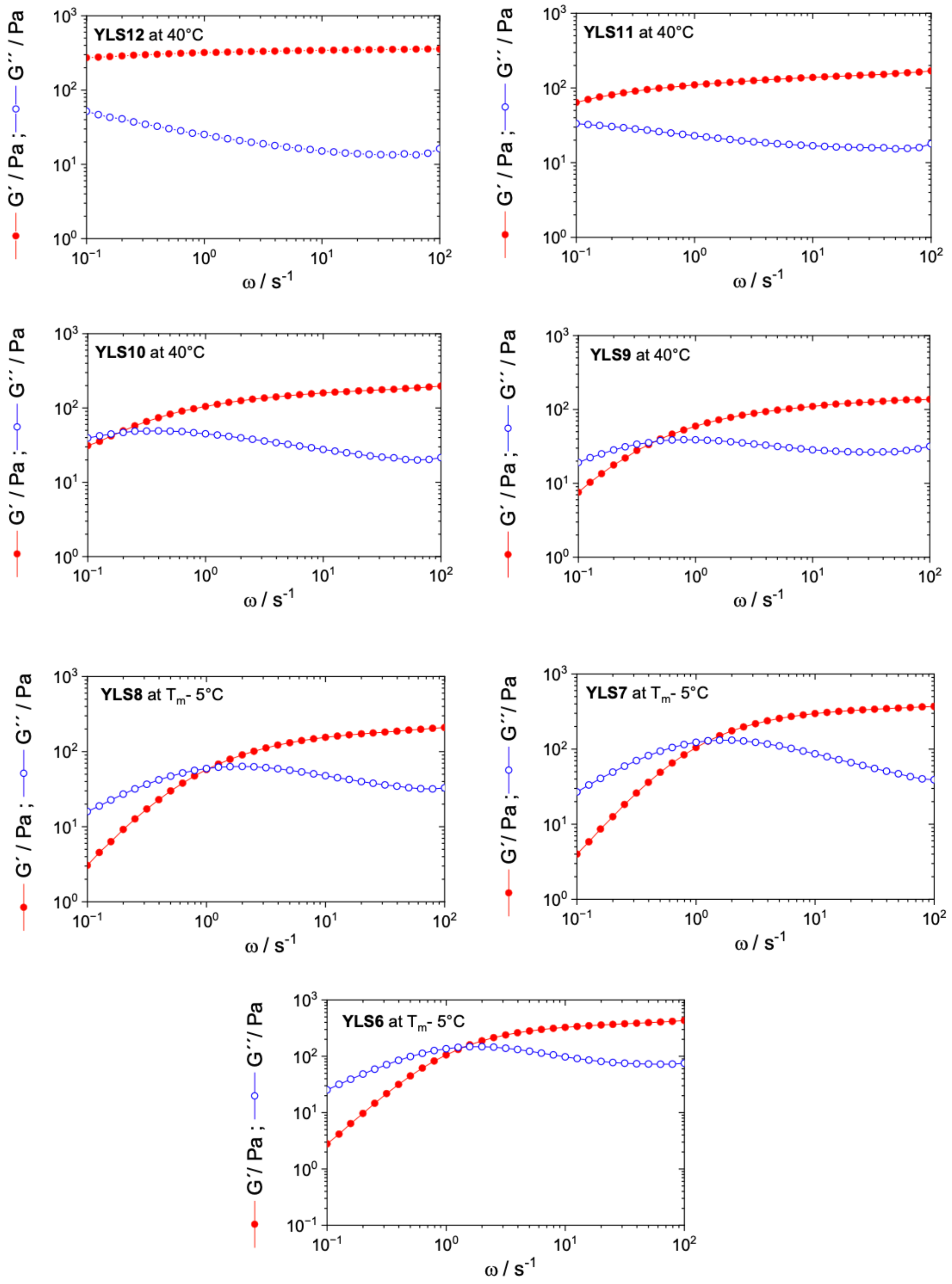

Supplementary Figure S 8: Frequency sweeps from bulk rheology on 1.5 wt% DNA hydrogels comprised of Y-shapes and linkers, where we compared YLS12, YLS11, YLS10 and YLS9 at 40°C, and YLS8, YLS7 and YLS6 at 5°C below their corresponding melting temperatures. We note the shift in relaxation time (inverse crossover of $G'(\omega)$ and $G''(\omega)$) on deleting bases from the sticky ends of the linkers and maintaining constant temperature. On the other hand, measuring at points equidistant from the melting transition, the relaxation times appear nearly identical.

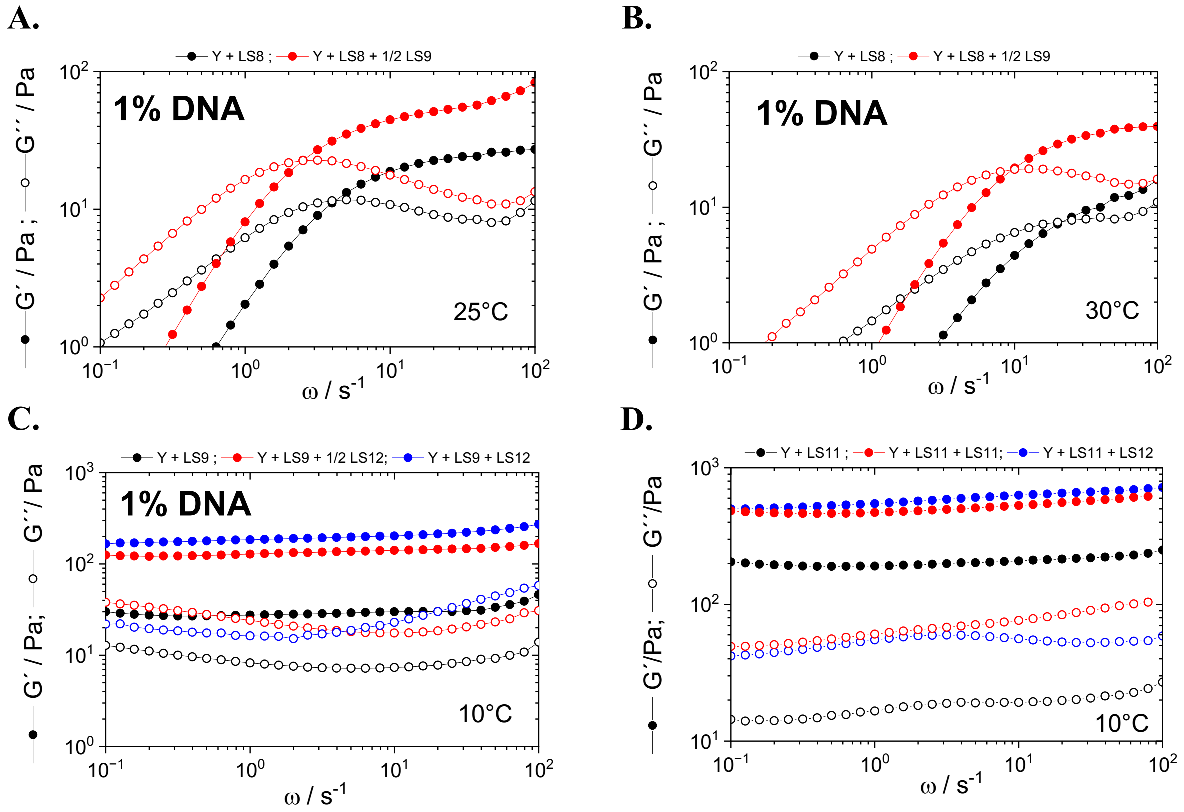

Supplementary Figure S 9: Linker-exchange experiments using bulk rheology on A)-C) 1 wt% DNA hydrogels or D) one-to-one ratio DNA hydrogels comprised of Y-shapes and linkers. A)-B) At temperatures of 25°C and 30°C, we started with measuring the rheology of YLS8, then adding LS9 linkers at half the concentration of LS8. We found notable upward shifts in both $G'(\omega)$ and $G''(\omega)$) on operating close to the the sol-gel transition of YLS8, which occurs at around 35-36°C (*cf.* Figure 6 of the main manuscript). C) Using the large difference in binding energy between YLS9 and YLS12, we were able to detect linker exchange even much below the sol-gel transition of YLS9 at a temperature of 10°C. D) Apart from detecting different concentrations of target linkers, we were also able to detect single-nucleotide variants by preparing 1:1 Y-shape:linker YLS11 hydrogel and adding either LS11 or LS12 at a temperature of 10°C. All of these experiments corroborate the linker-exchange findings in our main manuscript (*cf.* Figure 8).

5 Multi-particle tracking

To perform further structural characterisation of our sample, we used multi-particle tracking (MPT). In Supplementary Figure S11 we partially validated the indirect determination of 25-30 nm pore size from bulk rheology measurements. Here we used 200-nm PEG-ylated polystyrene particles as tracers. The figure shows first the trajectory of each tracer bead, showcasing that all beads were immobilised when embedded in a matrix of 1.5 wt% YLS11 at 20°C. The fluctuations in the trajectory of a single bead were on the order of 50 nm (*cf.* the second panel). Analysing the variation of the mean-squared displacement (MSD) as a function of lag time (last panel), we found virtually no time dependence (slope ≈ 0), confirming that the beads were indeed trapped within the hydrogel. The average MSD (red line) yields a value of around 7.5×10^{-4} μm^2. This result has a relatively narrow distribution of absolute MSD values, with a non-Gaussian parameter $\alpha < 1$ ($\alpha \approx 0.8$ @ t = 0.1 s). This suggests that all particles were trapped in a fairly uniform, homogeneous elastic network, whose mesh size is smaller than the diameter of the tracers (200 nm). The blue line represents the theoretical MSD variation of 200-nm diameter particle in water at 20°C, serving as guide to the eye.

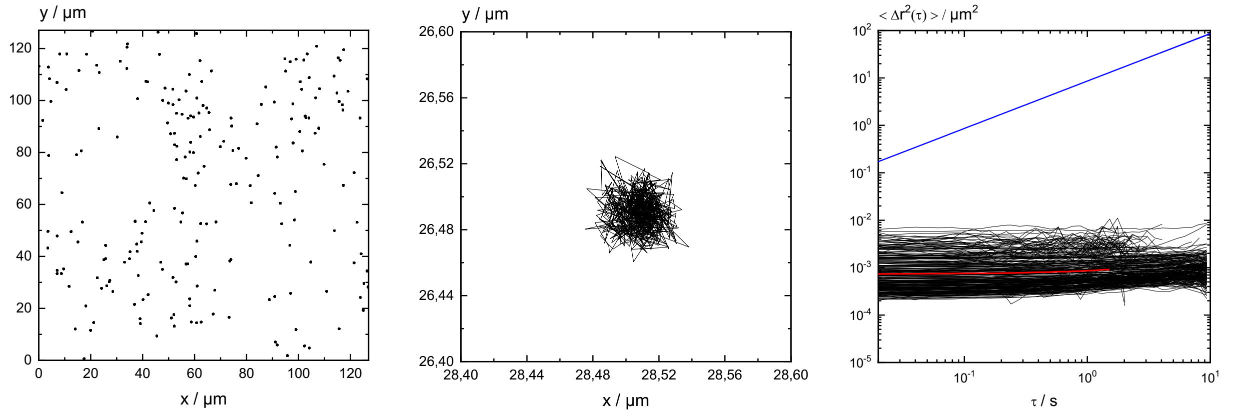

Supplementary Figure S 10: Multi-particle tracking of 200-nm PS-PEG particles in 1.5 wt% YLS11 hydrogel at 20°C. We observe nearly complete immobilisation of the tracers, suggesting the presence of a nearly homogeneous mesh with pore size well below 200 nm. The red line in the MSD plot indicates an estimated average of 250 particle trajectories and the blue line shows the diffusion of 200-nm PS-PEG bead in water, serving as reference. The data were collected at 50 frames per second, 500 images.

6 Cryo scanning electron microscopy

Finally, we attempted to use environmental SEM (ESEM, Thermo Fisher Scientific Quattro S) equipped with a cryogenic sample holder (Leica) to visualise the porous structure of our hydrogel. We combined 10 μL of Y-shapes (400 μM) and 10 μL of LS12 (600 μM) to form a 20-μL droplet of YLS12 hydrogel at room temperature (over 30°C below the measured sol-gel transition temperature). Then, we froze the droplet using liquid nitrogen in a cryo loading station (Leica EM VCM) and used a vacuum cryo-transfer system to subsequently transfer the sample to the ESEM. This measurement confirmed our findings from MPT that the microstruc-

ture of our hydrogels is highly porous and homogeneous. The pore size we obtained was rather large, which is possibly an artifact originating from the freezing process and can be partially attributed to the expansion of water on cooling. Additionally, the gelation process along the volume of the droplet was possibly heterogeneous due to the diffusion-limited gradient of mixing between the two DNA components. We expect that on the surface the mixing likely differs from that inside the droplet. We noted that stirring accelerates the gelation of the droplet, suggesting that the formation of the porous gel structure is limited by diffusion, and not by gelation rate. In the images below we only have access to the pores exposed on the surface, and we have no direct feedback on their appearance inside the droplet. For that, we would need to cut the frozen gel drop open and then perform cryo-SEM.

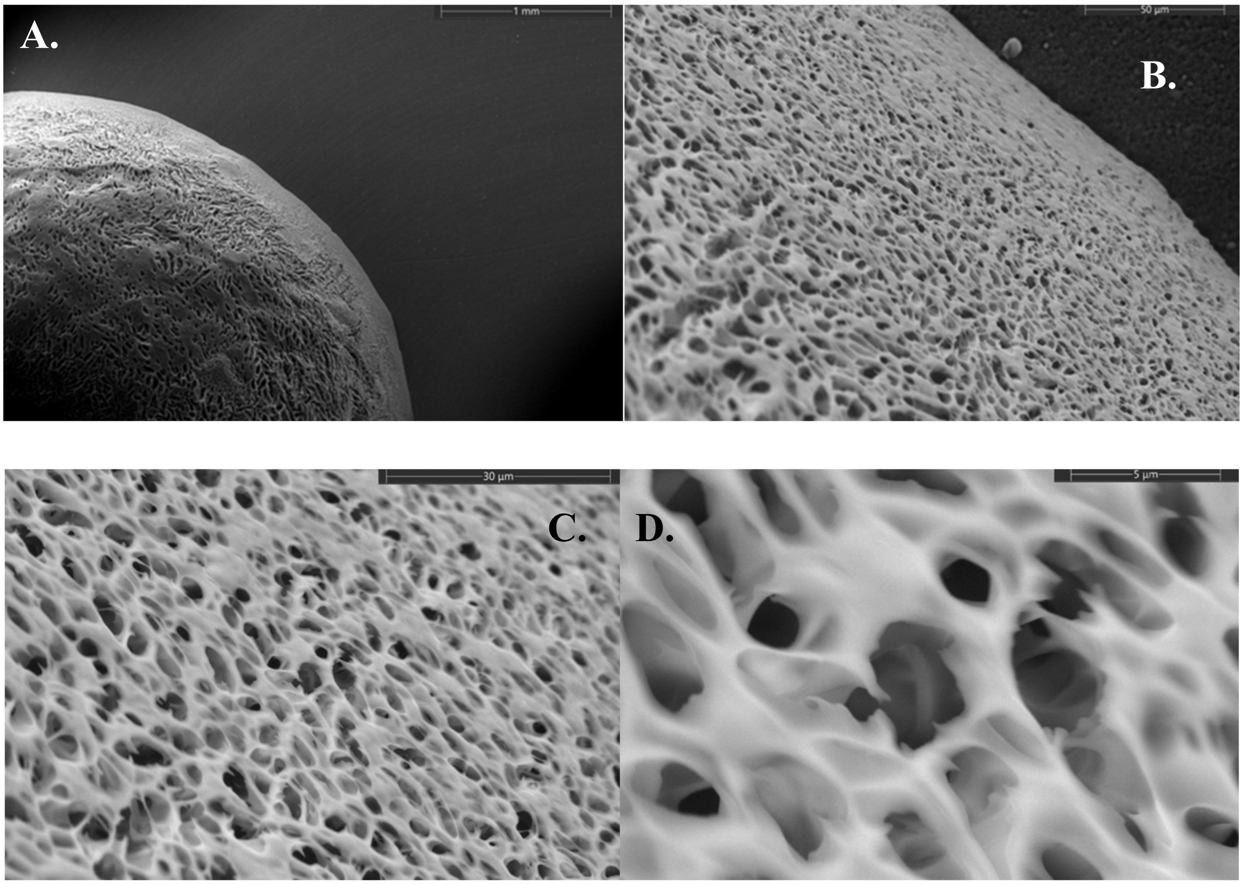

Supplementary Figure S 11: Cryo scanning electron microscopy of a droplet of 1.5 wt% YLS12 hydrogel. The snapshots show clearly the porous and homogeneous surface structure of the gel droplet. Scale bars: A) 1 mm, B) 50 μm, C) 30 μm, and D) 5 μm.
